# Faecal proteomics as a novel method to study mammalian behaviour and physiology

**DOI:** 10.1111/1755-0998.13380

**Published:** 2021-04-08

**Authors:** Takumi Tsutaya, Meaghan Mackie, Rikai Sawafuji, Takako Miyabe‐Nishiwaki, Jesper V. Olsen, Enrico Cappellini

**Affiliations:** ^1^ Department of Evolutionary Studies of Biosystems The Graduate University for Advanced Studies Hayama Japan; ^2^ Biogeochemistry Research Center Japan Agency for Marine‐Earth Science and Technology Yokosuka Japan; ^3^ Evolutionary Genomics Section The Globe Institute University of Copenhagen Copenhagen Denmark; ^4^ Proteomics Program Novo Nordisk Foundation Center for Protein Research Faculty of Health Science University of Copenhagen Copenhagen Denmark; ^5^ Primate Research Institute Kyoto University Inuyama Japan

**Keywords:** breastfeeding and weaning, diet, intestinal condition, Japanese macaque, proteomics

## Abstract

Mammalian faeces can be collected noninvasively during field research and provide valuable information on the ecology and evolution of the source individuals. Undigested food remains, genome/metagenome, steroid hormones, and stable isotopes obtained from faecal samples provide evidence on diet, host/symbiont genetics, and physiological status of the individuals. However, proteins in mammalian faeces have hardly been studied, which hinders the molecular investigations into the behaviour and physiology of the source individuals. Here, we apply mass spectrometry‐based proteomics to faecal samples (*n* = 10), collected from infant, juvenile, and adult captive Japanese macaques (*Macaca fuscata*), to describe the proteomes of the source individual, of the food it consumed, and its intestinal microbes. The results show that faecal proteomics is a useful method to: (i) investigate dietary changes along with breastfeeding and weaning, (ii) reveal the taxonomic and histological origin of the food items consumed, and (iii) estimate physiological status inside intestinal tracts. These types of insights are difficult or impossible to obtain through other molecular approaches. Most mammalian species are facing extinction risk and there is an urgent need to obtain knowledge on their ecology and evolution for better conservation strategy. The faecal proteomics framework we present here is easily applicable to wild settings and other mammalian species, and provides direct evidence of their behaviour and physiology.

## 1 INTRODUCTION

Faeces is a valuable material for studying the ecology and evolution of mammals. Faeces can be collected noninvasively during field research activities, and its availability is greater than other samples such as serum, muscle, or skeleton, in most field settings. Several kinds of analytical methods for faeces provide complementary evidence compared to conventional behavioural observation or invasive methods. For example, the observation of undigested food remains in faeces provides evidence of dietary intake (Gales & Cheal, 1992; Moreno‐Black, 1978). Genomic and metagenomic analyses of faeces reveal host genetics, taxonomy, diet, parasites, and intestinal flora (Barba et al., 2014; Hicks et al., 2018; Srivathsan et al., 2016). Stress and reproductive status are estimated from faeces by analysing the steroid hormones (Lasley & Kirkpatrick, 1991; Palme, 2005). Stable isotope analysis of faeces is utilized to estimate diet and weaning patterns (Bădescu et al., 2017; Blumenthal et al., 2012). Since faeces is composed of molecules originating from the host, ingested foods, and intestinal symbionts, it offers direct evidence of several aspects of different life phenomena.

This study focuses on proteins in faeces. Proteins are the functional entity of organisms, and their amino acid sequence or expression profiles can differ in different species and/or different tissues. Therefore, the analysis of proteins in faeces provides complimentary or more detailed knowledge on behaviour, physiology, and phylogeny of the studied mammalian individuals compared to those of other molecules. Some of this knowledge is difficult to retrieve when using other biomolecules, such as DNA, hormones, and stable isotopes. Compared to other molecules, proteins in faeces have not been fully investigated until recently. This is because the conventional methods to analyse proteins, such as immunoassay, are not suitable for the analysis of degraded proteins in a complex mixture of other proteins (Child & Pollard, 1992), which is at least partially true for those found in faeces. Protein detection by conventional immunological methods suffers from lower sensitivity and specificity, requires determination of target proteins before analysis, and does not work when the epitope is missing. While excreted proteins were successfully detected from faeces of human infants by immunoassay (Davidson & Lönnerdal, 1987; Goldman & Goldbaum, 1995; Haneberg & Finne, 1974; Lönnerdal, 2003; Patton, 1994), these previous studies are not reaching the full potential of faecal proteomics.

The recent development of mass spectrometry (MS)‐based proteomics now allows for detailed protein analysis of mammalian faeces. It enables accurate sequencing of any protein residue present in femtomolar quantities with no need to predefine a target (Aebersold & Mann, 2016). MS‐based proteomics is instead a more suitable method to analyse host, dietary, and symbiont proteins from faecal samples, even if they are partially digested and in small amounts. Although the MS‐based proteomics of faeces has been applied to detect clinical biomarkers of inflammatory bowel diseases in humans (Jin et al., 2017; Lehman et al., 2019) and metaproteomic analysis of human gut microbiota (Lee et al., 2017), as far as the authors are aware it has not been applied to research in ecology and evolution.

In this study, we present a framework of faecal proteomics as a novel method to study the behaviour and physiology of mammalian species. MS‐based proteomics was applied to 10 faecal samples collected from captive Japanese macaques (*Macaca fuscata*) in different stages of life history, and the proteomic results were evaluated with the known behavioural and physiological information of these individuals. Although the results we present do not expand the current knowledge about the behaviour and physiology of Japanese macaques, they are used to validate the reliability of faecal proteomics as an innovative approach to investigate mammalian ecology and physiology. Host proteins were the main target of this study, but the metaproteome originating from foods and intestinal microbes were also analysed. The results show that faecal proteomics is a useful method to: (i) investigate dietary changes related to breastfeeding and weaning, (ii) reveal the taxonomic and histologic origin of the food consumed, and (iii) characterize the gastrointestinal physiological status. These types of insights are difficult or impossible to obtain through other molecular approaches.

## 2 MATERIALS AND METHODS

### 2.1 Sample collection

**FIGURE 1:**
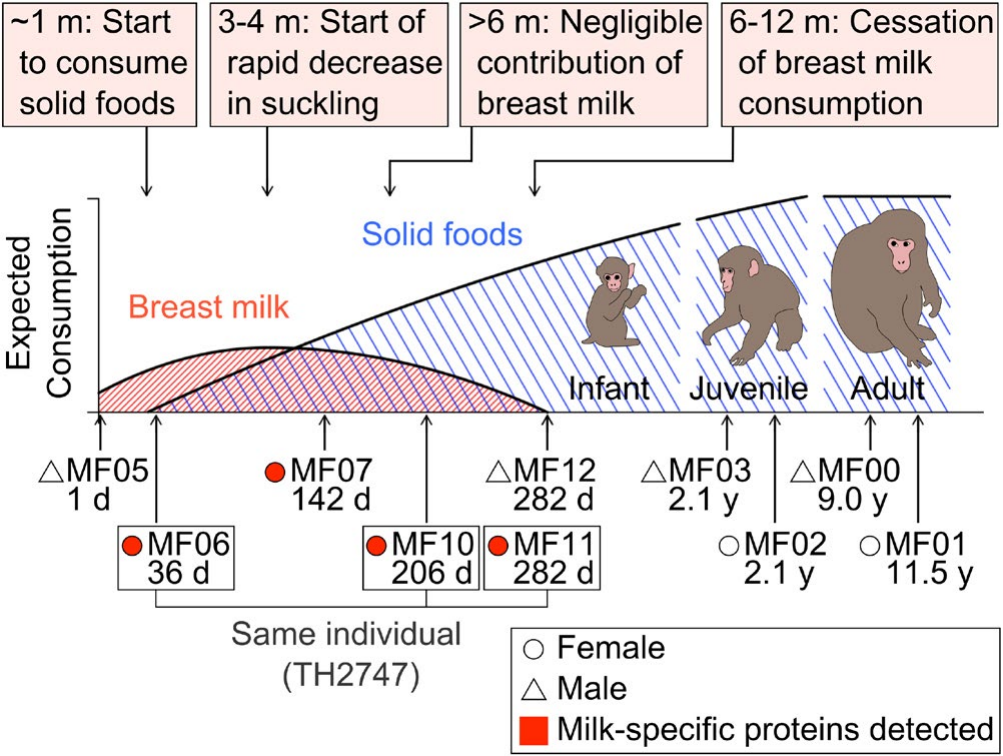
Schematic illustration of known breastfeeding and weaning patterns in Japanese macaques (see Supporting Information for more details) and the details of the analysed faecal samples (see Table S1 for more details)

A total of 10 faecal samples were collected from captive Japanese macaques kept at the Primate Research Institute, Kyoto University, Japan (Figure 1; Table S1). At the Institute, mother and infant (<1 year old) pairs are kept together in individual cages, and juvenile (1−4 years old) and adult (≥5 years old) individuals are kept in groups. The definition of age categories reported in MacIntosh et al. (2012) was used in this study. Juveniles are kept separately from their mother after 1−2 years old. The dropped faeces was collected from the cage along with the individual’s information, and stored in a freezer at −20°C until proteomic analysis. The macaques’ diet mainly consists of monkey pellets (“AS” produced by Oriental Yeast Co., Ltd.), and occasionally sweet potatoes. The major ingredients of the monkey pellets are rice, soybean, maize, wheat, and peanuts. This study was conducted following the Guide for the Care and Use of Laboratory Primates, after the approval of the Animal Care and Use Committee of Kyoto University (2017‐156, 2018‐093).

It can be estimated that infant and adult faecal samples represent dietary inputs during the latest 23−28 h and 32−60 h before defaecation, respectively, from experimental evidence of the mean retention time of low‐fibre dietary markers in Japanese macaques (Sawada et al., 2011, 2012). The faecal sample collected from an individual aged 1 day was greenish‐black meconium which represents intestinal proteins accumulated in the intrauterine environment (Lisowska‐Myjak et al., 2019; Figure S1). Breastfeeding and weaning patterns in captive Japanese macaques have been well studied through observational methods (Figure 1; Supporting Information), and such knowledge was referred to in this study.

### 2.2 Proteomic analysis

Since faeces is a complex material consisting of host tissues (including breast milk proteins), intestinal microbiota, and food remains, proteins were extracted using a modified method focused on host proteome characterization proposed by Lichtman et al. (2013). The bacterial fraction of faeces, containing a large amount of bacterial proteins, inhibits the detection of the relatively smaller amount of host proteins during the mass spectrometric analysis, and most of them were removed by centrifuge in this method (Lichtman et al., 2013). This method is expected to increase the detectability of proteins originating from the source individual and its foods, compared with other extraction methods that focus on the bacterial proteome of faeces (Lee et al., 2017). Details of the proteomic methodology are reported as Supporting Information. Briefly, faeces were disrupted in a guanidine hydrochloride solution and centrifuged to pellet insoluble materials. The supernatant was then ultracentrifuged to pellet bacteria. The supernatant samples, containing the soluble proteins, were then reduced and alkylated (adapted from Sawafuji et al., 2017; Tsutaya et al., 2019), and the protein solutions were then digested using trypsin overnight at 37°C. Tryptic peptides were purified using Stage Tips with C18 membrane (Rappsilber et al., 2007) and analysed by nanoflow liquid chromatography‐tandem mass spectrometry (nLC‐MS/MS), using an EASY‐nLC 1200 connected to a Q‐Exactive HF‐X (ThermoFisher).

RAW data files generated by LC‐MS/MS were searched against a database consisting of the rhesus macaque (*Macaca mulatta*) Uniprot proteome downloaded on 2019‐06‐28 and all available bacterial and relevant food (i.e., sweet potato, rice, soybean, maize, wheat, and peanuts) Swiss‐Prot proteomes downloaded on 2019‐06‐20 from Uniprot, as well as a common laboratory contaminant database provided with the MaxQuant software version 1.5.3.30 (Cox & Mann, 2008; Tyanova et al., 2016). Protein groups with at least two different nonoverlapping peptides were considered confidently identified, and MS/MS spectra were often manually examined for correct identifications. All protein hits that could be considered possible contamination products were excluded from further analysis. Detected proteins were classified using PANTHER database version 13.1 (Mi et al., 2013). The RAW data of the rhesus macaque milk proteome reported by Beck et al. (2015) was also analysed, for a comparative purpose, with the same procedures against the rhesus macaque proteome database (Table S2). All statistical analysis was performed on R software, version 3.6.1, or higher (R Core Team, 2020).

## 3 RESULTS

### 3.1 General profile of the faecal proteome

Excluding contaminants (e.g., human keratins and common laboratory reagents), a total of 741 (425 macaque, 76 food, and 240 bacterial) protein groups were detected from the 10 faecal samples of captive Japanese macaques (Table S3). RAW data have been uploaded to ProteomeXchange (Perez‐Riverol et al., 2019) with the data set identifier PXD021098.

**FIGURE 2:**
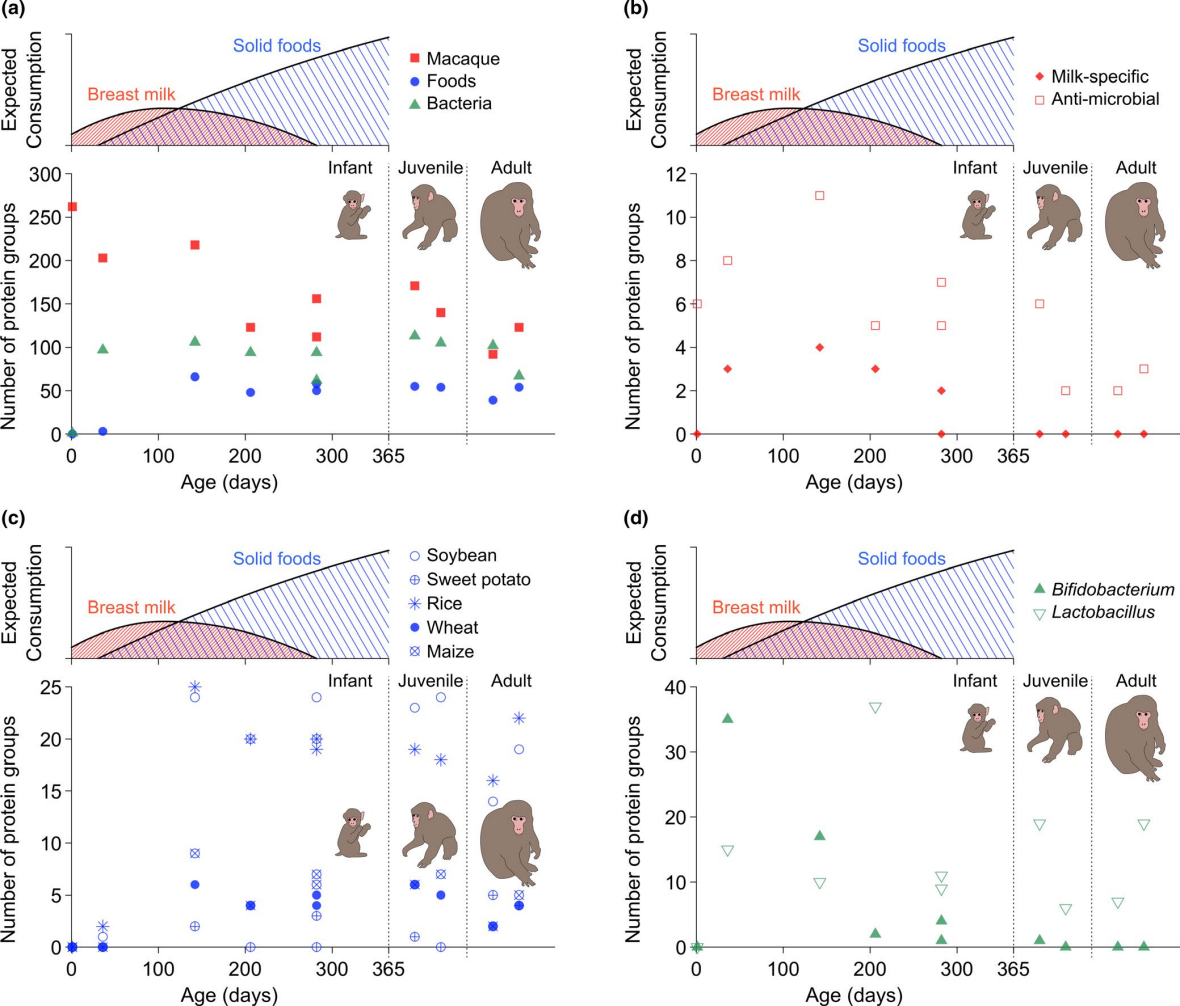
Number of protein groups detected from Faecal samples by the age of the individuals at the time of sample collection. Schematic illustrations of the amount of consumed breast milk and solid foods during infancy are also shown. (a) Number of macaque, food, and bacterial protein groups. (b) Number of milk‐specific and antimicrobial protein groups. (c) Number of food protein groups. (d) Number of *Bifidobacterium* and *Lactobacillus* protein groups

Although their confirmation by statistical tests is not possible due to the small sample size, the differences in an individual’s age show different patterns in the number of detected protein groups for the different classifications of proteins (i.e., macaque, food, and bacteria) (Figure 2a; Figure S2). A greater number of macaque protein groups were identified in younger individuals. On the other hand, the number of food protein groups increased from 0 at 1 day old to three at 36 days old, and the number of identified food protein groups became stable (53.0 ± 7.8, *n* = 8) after 142 days. Only one bacterial protein group was detected from the faecal sample at 1 day, but the number of identified bacterial protein groups also became stable (93.3 ± 17.5, *n* = 9) after 36 days old.

Venn diagrams show that unique macaque protein groups were found from the faeces of the youngest individuals (MF05, aged 1 day old, and MF06, aged 36 days old) compared with those of older individuals (Figure S2a). A greater number of detected macaque protein groups in the youngest individuals is also apparent (Figure S2d). However, PANTHER analysis (Mi et al., 2013) of macaque protein groups shows a functional similarity among the different age categories (Figure S3), suggesting the abundant unique macaque protein groups in the faeces of the youngest age category have similar functions compared with the common protein groups of all individuals.

### 3.2 Proteins originating from breast milk

Proteins that are expressed exclusively in breast milk (milk‐specific proteins) were identified only from infant faecal samples and not from juvenile and adult samples (Figure 2b; Figure S4). The identified milk‐specific protein groups are β‐casein, κ‐casein, α‐lactalbumin, and β‐lactoglobulin‐like protein (Table 1). Protein BLAST searches indicated that all recovered peptide sequences of these milk‐specific proteins match rhesus macaque proteins and do not derive from bovine milk proteins that can be used in laboratory reagents (Table S4). The score of most peptide‐spectrum matches for these milk‐specific proteins is also high (Figure S5), as confirmed by manual observation.

**TABLE 1:** Milk‐specific, potential breast milk marker, antimicrobial, and age‐dependent protein groups detected from faecal samples. Number of identified razor + unique peptides is also shown

Classification	Gene	Protein	Protein ID	Sequence coverage	Score	*N*. peptide
Milk‐specific	CSN2	Beta‐casein	F6UED1	28.3	323.31	5
	CSN3	Kappa‐casein	F6WJ58	17.0	31.73	2
	LALBA	Lactalbumin alpha	F6X0Y7	26.8	244.31	5
	LOC721853	Lactogrobulin‐like	F6YKN5	41.2	36.45	5
Potential marker	FABP3	Fatty acid binding protein 3	G7MI71	64.7	323.31	8
	PLIN2	Perilipin	F7GZC0	16.4	323.31	2
	LPO	Lactoperoxidase	F7BAC5	4.1	22.139	6
Anti‐microbial	B2M	Beta‐2‐microglobulin	A0A1D5Q7X4	30.9	73.79	3
	C2	Uncharacterized protein	F7D5J9	3.0	11.71	2
	C3	Complement C3	F7EV32	23.2	323.31	28
	C9	Complement C9	F7GRY2	3.6	11.76	2
	IGHM	Immunoglobulin heavy constant mu	A0A1D5Q3R9	27.3	202.60	12
	JCHAIN	Immunoglobulin J chain precursor	A0A1D5RFA2	29.0	88.42	8
	LCN2	Lipocalin 2	H9H4D4	72.7	323.31	17
	LPO	Lactoperoxidase	F7BAC5	4.1	22.14	2
	LTF	Lactotransferrin isoform 1	F7BYZ5	58.6	323.31	46
	LYZ	Lysozyme C	P30201	16.2	13.75	2
	MUC1	Mucin 1, cell surface associated	A0A1D5Q9X6	14.1	81.36	2
	PIGR	Polymeric immunoglobulin receptor	F6WY82	22.9	279.18	17
Age‐dependent	LCT	Lactase	F6V0B1	18.2	323.31	37

Although its confirmation by statistical tests is not possible due to the small sample size, the total number of milk‐specific protein groups identified showed an age‐dependent decline along with their breastfeeding and weaning patterns (Figure 2b). Milk‐specific proteins were not detected from faeces collected at 1 day after birth. At least two milk‐specific protein groups were identified from faecal samples collected from individuals aged from 36 to 282 days old, except for one sample collected at 282 days after birth (MF12). Since juveniles and adults older than two years are completely weaned, milk‐specific proteins are not detected from their faeces (Figure 2b).

**FIGURE 3:**
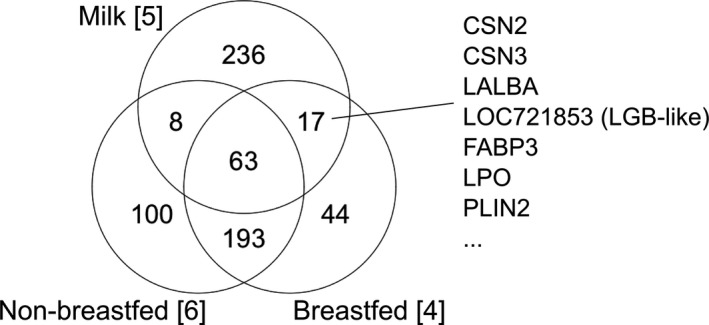
Venn diagrams of the macaque protein groups detected from breast milk (Beck et al., 2015), faeces of breastfed individuals (MF06, MF07, MF10, and MF11), and faeces from nonbreastfed individuals (MF05, MF12, MF03, MF02, MF00, and MF01). See Table 1 for the detailed information of the milk proteins shown

There are 17 protein groups, relating to milk and faecal proteomes, that are exclusively present in the samples from the breastfed individuals (Figure 3; Supporting Information). All four milk‐specific proteins were included here, as well as lactoperoxidase (LPO), perilipin 2 (PLIN2), and fatty acid binding protein 3 (FABP3) (Figure S4; Table 2). They are expressed in other body fluids, but present with a relatively higher concentration in macaque breast milk (Beck et al., 2015).

**TABLE 2:** Food protein groups that indicate the derived plant organ/tissue detected from faecal samples. Number of identified razor + unique peptides is also shown

Species	Gene	Protein	Protein ID	Sequence coverage	Score	*N*. peptide
*Glycine max*	BMY1	Beta‐amylase	P10538	3.6	11.624	2
	LOX1.1	Seed linoleate 13S‐lipoxygenase‐1	P08170	13.2	166.76	10
	LOX1.2	Seed linoleate 9S‐lipoxygenase‐2	P09439	10.2	54.676	7
	LOX1.3	Seed linoleate 9S‐lipoxygenase‐3	P09186	10	178.2	7
	SBP65	Seed biotin‐containing protein SBP65	Q39846	6.5	25.094	4
*Ipomoea batatas*	BMY1	Beta‐amylase	P10537	47.1	323.31	19
	GSPO‐A1	Sporamin A	P10917	13.2	42.832	2
	GSPO‐B1	Sporamin B	P10965	52.3	219.32	8
	−	Sporamin A	P14715	31.5	165.42	4
*Oryza sativa*	GLUA3	Glutelin type‐A 3	Q09151	39.3	323.31	20
	GLUB2	Glutelin type‐B 2	Q02897	19.6	113.61	5
	GLUB5	Glutelin type‐B 5	Q6ERU3	57.6	323.31	31
	GLUD1	Glutelin type‐D 1	Q6K508	14.3	30.975	5
	Os03g0793700	Cupincin	Q852L2	7.9	18.976	2
	Os12g0269200	Prolamin PPROL 17D	P20698	50	323.31	4
	PROLM20	Prolamin PPROL 14P	Q42465	55.6	323.31	5
	PROLM25	13 kDa prolamin C	P17048	48.1	323.31	4
	PROLM7	Prolamin PPROL 14E	Q0DJ45	52.7	323.31	5
	RA5	Seed allergenic protein RA5	Q01881	53.8	67.861	7

### 3.3 Proteins originating from food items

Protein groups that indicate the derived plant organ/tissue, as well as biological species, were recovered from the faecal samples analysed in this study (Table 2). The number of food protein groups detected is smaller (0 and 3) in the faecal samples from the youngest individuals (1 and 36 days old), compared with that from the older individuals (53.0 ± 7.8, *n* = 8; Figure 2a, Figure S2e), suggesting the amount of solid foods consumed up to 36 days old was little to none. Protein groups of all the plants that are the ingredients of the monkey pellets (i.e., rice, soybean, maize, wheat, and peanuts) and fed to the macaques by the keepers (i.e., sweet potato) were identified from the faecal samples, except for peanuts (Table S3). In this small sample set, changes in the number of detected protein groups of different plant species show consistent age‐dependent patterns (Figure 2c).

### 3.4 Proteins representing the physiological status of the host

Breast milk, as well as other body fluids, contains a wealth of antimicrobial proteins (Goldman & Goldbaum, 1995; Lönnerdal, 2003), and 12 protein groups that work as direct‐acting antimicrobial agents were detected from the faecal samples analysed in this study (Table 1; Supporting Information). The number of detected antimicrobial protein groups was greater in the infant samples compared to those from the adults, and their relationship with an individual’s age is similar to that of milk‐specific proteins (Figure 2b), although confirmation by statistical tests is not possible due to the small sample size. The number of detected antimicrobial protein groups increases after birth, reaches its peak at 142 days old, and decreases thereafter, remaining still detectable from the faeces of juveniles and adults (Figure 2b, Figure S6).

The detection of lactase (LCT) shows an age‐dependent expression in the intestinal environment, related to the host’s dietary development. It was detected only from faecal samples collected from infants and juveniles, except for a faecal sample collected at 1 day after birth (Table 1; Figure S6). Lactase is an essential enzyme needed to metabolize lactose in breast milk, and its expression ceases after the end of weaning process in most mammalian species (McCracken, 1971).

Only one bacterial protein group was detected from faeces of the youngest individual (1 day old) while a greater number of bacterial protein groups (93.3 ± 17.5, *n* = 9) was detected in faeces of older individuals (Figure 2a; Table S3). The number of intestinal bacteria starts to increase a few days after birth, and *Bifidobacterium*, a major bacterial taxon that is included in breast milk and metabolizes oligosaccharides, becomes dominant in breastfed infants (Fanaro et al., 2003; Mueller et al., 2015; Rhoades et al., 2019; Tamburini et al., 2016). The number of *Bifidobacterium* protein groups detected was greater in younger individuals (35 and 17 in individuals aged 36 and 142 days old), but rapidly decreased (1.1 ± 1.5, *n* = 7) in samples from older individuals (Figure 2d; Tables S3 and S5).

## 4 DISCUSSION

This study aims to show the usefulness of faecal proteomics for investigating dietary changes and content, as well as characterizing the gastrointestinal status of the individuals studied. In order to achieve this goal, the results are discussed below in terms of authenticity, identity, and interpretation, as well as future possibilities and limitations.

### 4.1 Authenticity of the recovered faecal proteome

Proteins are ubiquitous molecules, and thus assurance of the endogeneity of the recovered proteome is essential for correct interpretations, especially when the target proteins are partially degraded (Hendy, Welker, et al., 2018a). Although there is a possibility of environmental and technical contamination of exogenous proteins in the recovered proteome, empirical evidence obtained in this study indicates that such a possibility is minimal. Age‐dependent changes (e.g., detectability of LCT and the number of milk‐specific, food, and bacterial proteins) of the recovered faecal proteomes are consistent with the identity of the host individuals (e.g., dietary status and age). The protein groups of interest were additionally not detected from experimental blanks. Furthermore, protein BLAST searches confidently showed that the detected peptides of milk‐specific proteins originated from macaque (Table S4), and not from bovine milk that can be used in laboratory reagents. This evidence supports the authentic endogenous origin of the faecal proteome retrieved.

Age‐dependent changes in the digestive ability and the concentration of proteins in breast milk seem to generate no systematic bias in the number of detectable milk‐specific protein groups from infant faeces during the weaning process (Supporting Information), which is important for the comparison between individuals of different ages in this study. While the digestive ability of an individual experiences an approximately two‐fold increase from the start to the end of weaning (Agunod et al., 1969; Bujanover et al., 1988; Deren, 1971; Walthall et al., 2005), the proportion of milk protein in breast milk also experiences an approximately two‐fold increase during the same period (Ôta et al., 1991; Hinde et al., 2009). These factors probably counteract each other, and a chronological pattern of the number of milk‐specific proteins detected in faeces would mostly reflect a degree of breast milk consumption. However, the number of food proteins detected in infants would be overestimated while its numbers are still smaller or similar compared with juveniles and adults (Figure 2a). Digestive efficiency becomes stable after 1 year of age (Sawada et al., 2012), which results in no systematic bias in juveniles and adults.

### 4.2 Identified proteins from the faecal samples

Milk‐specific proteins (i.e., β‐casein, κ‐casein, α‐lactalbumin, and β‐lactoglobulin‐like protein) were identified only from breastfed individuals (Table 1; Figure 2b; Figure S4). Caseins are the major milk proteins that comprise approximately 33% of the major protein fraction in human milk and provide proteinaceous nutrients to infants (Conti et al., 2007). Caseins form a micelle, and its digested peptides have several bioactive roles in gastrointestinal environments (Holt et al., 2013; Shah, 2000). Alpha‐lactalbumin is a calcium‐binding whey protein that is involved in the biosynthesis of lactose in the mammary gland (Permyakov & Berliner, 2000), which comprises approximately 28% of the major protein fraction in human milk (Conti et al., 2007). Beta‐lactoglobulin (LGB) is a major whey protein absent in some primate species, including humans and chimpanzees (Kontopidis et al., 2004). Although its exact physiological function is not yet determined, β‐lactoglobulin binds several hydrophobic ligands and thus may act as a specific transporter (Kontopidis et al., 2004).

There are several interesting aspects in the age‐dependent change of the total number of milk‐specific proteins along with the breastfeeding and weaning patterns (Figure 2b). No milk‐specific proteins were detected in the faeces collected at 1 day after birth. This suggests that breastfeeding had not started at the moment of sample collection from this individual or that at least the consumed breast milk had not yet passed through the intestinal tract, which usually takes 23−28 h (Sawada et al., 2012). The faecal sample collected at 1 day after birth is actually the meconium, and therefore slightly different protein composition compared with other faecal samples collected from older ages (Figure S2) was expected. Meconium does not contain intestinal microbiota or consumed foods, and instead represents proteins accumulated in the intrauterine environment (Lisowska‐Myjak et al., 2019). Among the faecal samples collected from individuals aged 282 days old, one (MF11) contained two milk‐specific protein groups but the other (MF12) contained no milk‐specific protein (Figures 1, 2b; Figure S4). This discrepancy might indicate an individual difference in breastfeeding and weaning patterns in macaques (Hinde, 2009; Hinde et al., 2009; Reitsema et al., 2015).

Faecal proteomics revealed information about the organ/tissue, as well as the taxonomy, of the consumed solid foods (Table 2). For example, sporamin is a major storage protein in tuberous roots, and accounts for 60%−80% of the total soluble protein in the sweet potato tuber (Yeh et al., 1997). Prolamin and glutelin are major storage proteins in seeds (Tan‐Wilson and Wilson, 2012). Linoleate lipoxygenases are involved in the degradation of storage lipids in oilseeds (Feussner et al., 2001). Beta‐amylase is involved in the metabolism of starch in plant tissues, such as seeds, tubers, and fruits (Beck & Ziegler, 1989). The reference food protein database used in this study consisted exclusively of the “correct” food species known to be included in the macaques’ diet. This is because the exploratory nature of this study aims to evaluate a novel analytical framework. However, taxonomic identification of food sources for individuals without a known dietary record is also possible by including proteins from candidate food species into the database as previously done for various materials (e.g., Hendy, Colonese, et al., 2018b; Mackie et al., 2018; Scott et al., 2021).

The detection of abundant antimicrobial proteins from faeces suggests that the intestinal environment of the host was well protected from microbial agents. However, it is not clear whether the high level of antimicrobial proteins in faeces simply represents a sufficient level of biological protection or is induced by an increased pathological load. Specific research should be designed to further investigate relationships between antimicrobial proteins and intestinal microbes. Nonetheless, the greater number of detected antimicrobial proteins in faecal samples collected from younger individuals would reflect maternal supply via breast milk in breastfed infants (Goldman & Goldbaum, 1995; Goldman et al., 1998; Lönnerdal, 2003) or placenta and amniotic fluid in the neonate (Jennewein et al., 2017).

Lactase (LCT) was detected from faecal samples collected from infants and juveniles (Table 1; Figure S6). Breast milk of Japanese macaques contains approximately 6.2% lactose (Ôta et al., 1991), which is subsequently metabolized by lactase expressed in the small intestine (McCracken, 1971). The expression of lactase ceases toward adulthood after the end of weaning in most mammals (McCracken, 1971), and the age‐dependent detectability of lactase in the macaque faeces agrees with this evidence (Figure S6). The detection of LCT in faeces suggests that the host individual is young and able to digest the lactose contained in maternal breast milk.

The number of detected bacterial protein groups is consistent with evidence derived from the metagenomic studies of breastfeeding and weaning‐related developments in human and macaque intestinal microbiota (Fanaro et al., 2003; Rhoades et al., 2019). After the introduction of solid foods during the weaning process, the dominance of *Bifidobacterium* rapidly ceases and the proportions of other bacterial taxa increases (Fanaro et al., 2003; Rhoades et al., 2019). The higher number of *Bifidobacterium* protein groups in faeces indicates that the host individual’s intestinal flora was more adapted to a breast milk‐based diet. Although metagenomic evidence suggests a possible enrichment of *Lactobacillus* in breastfed infants (Tamburini et al., 2016), such a tendency was not seen in this study (Figure 2d).

### 4.3 Faecal proteome provides direct evidence on behaviour and physiology

The reconstructed patterns of breast milk and solid food consumption by faecal proteomics agree with the general breastfeeding and weaning patterns already observed in the Japanese and closely related rhesus macaques. The smaller number of detected food proteins (Figure 2c) and the greater number of detected protein groups from *Bifidobacterium* (Figure 2d) in the infant faecal sample collected at 36 days suggest that the initial introduction of solid foods was started after approximately 1 month of exclusive breastfeeding. Faecal proteomics revealed that the consumption of breast milk protein increases after birth, reaches its maximum when the individual is around four months old, and ceases when it is around 9.5 months old, with some individual variation, in captive Japanese macaques (Figure 2b; Figure S4). Behavioural, stable isotopic, and trace elemental studies have shown a similar pattern of breastfeeding and weaning patterns in captive Japanese and rhesus macaques (Austin et al., 2013; Bowman & Lee, 1995; Nigi, 1982; Ôta et al., 1991; Reitsema et al., 2015).

Breastfeeding and weaning behaviours are important life events in wild mammals (Hinde & Milligan, 2011; Lee, 1996; Sellen, 2007) and have been studied by behavioural observation or isotopic/elemental analyses. However, the amount of breast milk consumed by an infant cannot be estimated from observational data due to the inconsistency between time spent suckling and amount of ingested milk (Cameron, 1988; Scanlon et al., 2002), as well as nonobservable night‐time breastfeeding (Miller et al., 2013). It is also difficult to observe when infants begin to consume solid foods and the consumed food materials themselves, because they frequently mouth food materials for play without ingesting them (Watts, 1985). Analyses of stable isotopes and trace elements are alternative methods to estimate the age relationship with the amount of consumed breast milk in mammals (Tsutaya & Yoneda, 2015). However, stable isotope ratios of faeces contain a mixture of small signals (i.e., host elements that represent breast milk consumption) and large noise (i.e., solid foods and intestinal microbiota). Therefore, estimating a breastfeeding and weaning pattern using faecal stable isotope ratios requires hundreds of infant faecal specimens across the entire infancy to extract the true signal from the noise (Bădescu et al., 2017; Reitsema, 2012). Furthermore, isotopic/elemental proxies only provide indirect evidence of breast milk because stable isotope ratios and trace element concentrations are also affected by the diet and physiology of the individual (Austin et al., 2016; Reitsema, 2013).

Faecal proteomics is expected to be a more feasible and accurate method to estimate breastfeeding and weaning patterns of wild mammals and additionally provides direct evidence of solid foods if present. Mammalian faecal samples can be collected easily and noninvasively in most wild settings, and direct evidence of breastfeeding and weaning patterns can be obtained using proteomics from relatively smaller numbers of infant faecal samples, compared with the isotopic estimation using faeces (Bădescu et al., 2017; Reitsema, 2012). Indeed, faecal proteomics confidently identified milk proteins from two out of three faecal samples collected from infant individuals aged older than six months (i.e., 6.9 and 9.4 months old) (Figure 2b; Figure S4), and its detection rate is higher than other methods, while strong conclusions cannot be drawn due to the small sample size used in this study. The nutritional contribution of breast milk was estimated to be negligible after six months old in the previous behavioural observational studies of Japanese macaques (Nigi, 1982; Ôta et al., 1991), and four out of eight captive rhesus macaque individuals were estimated to be completely weaned by 6 months old, and 6 out of 8 by 8 months old, by previous stable isotope analysis of serum (Reitsema et al., 2015).

Mammalian species typically consume different parts of plants (e.g., fruits, leaves, flowers, or seeds) or prey in different developmental stages (e.g., larva, young leaves, or ripe fruits) as different food sources. The visual observation of undigested food objects in faeces is biased toward hard and indigestible objects (Gales & Cheal, 1992; Moreno‐Black, 1978). Therefore, improving the resolution of estimates of food items greatly benefits studies in feeding ecology. Faecal proteomics does not only provide information about the taxonomy but also the organ/tissue origin of the consumed solid foods (Table 2), which is difficult to obtain through metagenomic analysis of food remains in faeces (Mallott et al., 2017; Sawada et al., 2014; Srivathsan et al., 2016) and stable isotope analysis (Bădescu et al., 2017; Blumenthal et al., 2012). While the faecal samples used in this study contained no food remains that were identifiable from visual observation (Figure S1), taxonomy and derived organ/tissue of dietary items were successfully identified using proteomic analysis (Table 2). If protein markers are known for a specific organ/tissue or developmental stage of a consumed species, the consumption of these food items can be confidently identified by faecal proteomics.

Faecal proteomics also provides direct evidence on the physiological status of the host’s intestinal environments. Conversely, faecal DNA analysis only provides genetic information and indirect evidence on the behaviour or physiology of the studied organisms. Faecal hormone analysis can be used to estimate the physiological status of the host, but it is only applicable to certain aspects of life phenomena, such as stress and reproduction. Compared to these molecules, proteins in faeces can reveal complementary evidence. Diversity of antimicrobial proteins in faeces is an indicator of intestinal protection to microbial agents (Figure 2b), detectability of LCT in faeces can be used as a discriminant factor for the age class of the host, and the age‐dependent change in the number of *Bifidobacterium* proteins reveals the function of intestinal microbiota (Figure 2d). Clinical marker proteins for intestinal bowel diseases or colorectal cancer (Jin et al., 2017; Lehman et al., 2019) in faecal samples can be used for the diagnosis of diseases in mammalian individuals, although such marker proteins were not detected from faecal samples of the target individuals in this study. Furthermore, in‐depth analysis of microbial proteins in faeces can reveal functional interactions between host and symbionts as had been reported for dental calculus (Jersie‐Christensen et al., 2018; Warinner et al., 2014), while the experimental protocol used in this study is not directed toward microbial proteins (see Methods).

### 4.4 Future possibilities and limitations

Most mammalian species are facing human‐induced extinction risk (Ceballos et al., 2015) and there is an urgent need to obtain knowledge on their ecology and evolution for better conservation strategies. While the conventional ecological survey and behavioural observation can provide detailed knowledge, it is labour‐intensive and usually requires a lot of time. Instead, molecular analyses of faecal samples, that can be collected easily and noninvasively, offer a novel framework to the research of mammalian ecology and evolution, which produces complementary evidence and can potentially speed up the growth of natural history research (Srivathsan et al., 2019). Faecal proteomic methods developed in this study expand such a framework and provides direct evidence on breastfeeding and weaning patterns, diet, and physiological status of the intestinal environment. Since protein functions and defaecation are universal life phenomena, the developed methodology is easily applicable to other mammalian species and wild settings. Furthermore, proteins are relatively resistant to degradation and preserve better than other biomolecules, such as DNA, beyond millions of years (Cappellini et al., 2019; Demarchi et al., 2016; Welker et al., 2019). Although facilities for sample storage, such as freezers, are limited in most wild settings, proteomics would be a feasible method for faecal samples that were not frozen but dried and stored at ambient temperature on site.

However, there are two major challenges when applying this proteomic method to faecal samples obtained from free‐ranging individuals in the wild, in order to address questions in complex natural environments. First, there will be a greater risk of contamination from exogenous proteins, and the degradation of endogenous proteins will likely be higher under the suboptimal settings of sample collection and storage in the field. An experimental approach is needed to understand the effect of different conditions of sample collection and storage in faecal proteomics. Second, a larger sample size would be required in order to draw strong conclusions on the behaviour and physiology of the target species in the wild settings. Compared to captive settings, heterogeneity of diet, breadth of dietary items, and the complexity of behaviours and physiological status would be greater in most wild settings. Under such conditions, it is important to evaluate a sufficient sample size for unbiased descriptions of the faecal proteomes, as has been tested in environmental DNA studies (e.g., Grey et al., 2018).

Furthermore, faecal proteomics to study behaviour and physiology of mammals is also associated with some limitations. This method is difficult to apply to mammalian species whose reference proteome and/or genome is not yet publicly available. This is because bottom‐up proteomics methods often require a reference proteomic database to match the MS spectrum to a specific amino acid sequence (Elias & Gygi, 2007; Tyanova et al., 2016). The use of a reference proteome of a closely related species would be a partial solution for this problem. Indeed, most proteins in this study were successfully identified with the reference proteome of the rhesus macaque, when the sequence was not available for the Japanese macaque. Insufficient coverage of proteomic and genomic databases for food items and intestinal bacteria of wild mammalian species is also a limiting factor for the identification of food and bacterial proteins. Accumulation and expansion of sequence data for mammals, food items, and intestinal bacteria will enable a more detailed and broader investigation of behaviour and physiology in wild and captive mammalian species. Although the costs of shotgun proteomic analysis are still considerable (a few hundred Euros per sample), future methodological developments could significantly reduce the costs of this approach.

## AUTHOR CONTRIBUTIONS

T.T., M.M., T.M.‐N. and E.C. designed this study, collected most of the data, and wrote the manuscript, which was critically read and revised by all authors. M.M. and J.V.O. provided LC‐MS/MS analysis. T.T. and R.S. analysed data. All authors approved the final version of the manuscript.

## Supporting information

Figure S1

Table S2

Table S3

## Data Availability

The data sets generated during and/or analysed during the current study are available in the ProteomeXchange repository with the data set identifier PXD021098.
