# Supplementary material for: Faecal proteomics as a novel method to study mammalian behaviour and physiology
Source: Mol Ecol Resour. 2021 Apr 8;21(6):1808–19. doi: 10.1111/1755-0998.13380 (PMC8360081; doi:10.1111/1755-0998.13380)
Supplement: Supplementary file 1 — Figure S1 [file MEN-21-1808-s003.docx]

**Supplemental Information for:**

**Faecal proteomics as a novel method to study**

**mammalian behavior and physiology**

Takumi Tsutaya*, Meaghan Mackie, Rikai Sawafuji,

Takako Miyabe-Nishiwaki, Jesper V Olsen, Enrico Cappellini*

**Table of Contents:**

| 1. **Supplementary Materials and Methods**    1. Expected breastfeeding and weaning statuses in the target individuals    2. Protein extraction and proteomic analysis    3. Data analysis | Page 2 |
| --- | --- |
| 1. **Supplementary Results and Discussion**    1. Proteins that were found from experimental blanks    2. Potential protein marker of breast milk intake    3. Details of the detected anti-microbial proteins    4. Confounding factors for the proteomic estimation of breastfeeding and weaning patterns | Page 4 |
| 1. **References** | Page 6 |
| 1. **Supplementary Figures** | Page 9 |
| 1. **Supplementary Tables** | Page 23 |

**1. Supplementary Materials and Methods**

**1.1 Expected breastfeeding and weaning statuses in the target individuals**

Breastfeeding and weaning patterns in captive Japanese macaques have been well studied through observational methods (Nigi, 1982; Ôta et al., 1991). According to these previous observations, infants start to consume non-milk foods (i.e., start the weaning process) after the first month of age, frequency and duration of suckling behavior start to rapidly decrease around 3−4 months old, nutritional contribution of breast milk seems to be negligible after 6 months old, and cessation of breast milk consumption (i.e., end of weaning) occurs at 0.5−1 years old. Wild Japanese macaques follow similar breastfeeding and weaning patterns, except that the age at the end of weaning seems to be several months later than their captive counterparts (Hiraiwa, 1981; Tanaka, 1992). Also, stable isotopic, trace elemental, and behavioral studies have revealed the following weaning trajectory in captive rhesus macaques: nutritional contribution of solid foods greatly increases from 2 to 5−6 months, breast milk consumption ceases during 5−10 months, and the next conception of the mother starts around 7.4 months after birth (Austin et al., 2013; Bowman and Lee, 1995; Reitsema et al., 2015).

The behavioral breastfeeding and weaning status in the host individuals of the fecal samples analyzed in this study can be estimated as follows (see also Table 1). MF05, a sample collected from a 1 day old individual, would not contain breast milk proteins because his mother denied rearing him and the infant could not suckle his mother’s breast. MF06 was collected from an individual aged 36 days and reflects the final period of exclusive breastfeeding. MF07, collected from an individual aged 142 days, corresponds to the period when the individual suckles intensively but also consumed solid foods. MF10 (206 days) corresponds to the period of rapid decline in suckling frequency and duration. MF11 (282 days) and MF12 (282 days) were collected from individuals during the final period of the weaning process, and it is not clear whether these individuals consumed breast milk. Fecal samples collected from 2-year-old juveniles (MF03 and MF02) and adults (MF00 and MF01) should not contain breast milk proteins.

**1.2 Protein extraction and proteomic analysis**

Proteins were extracted from approximately 50−100 mg of Japanese macaque (*Macaca fuscata*) fecal samples by using a modified method of Lichtman et al. (2013) and Tsutaya et al. (2019), along with additional blank controls. Feces were suspended in 6 M guanidinium hydrochloride (GuHCl) solution containing 10 mM tris(2-carboxyethyl)phosphine hydrochloride (TCEP) and 20 mM 2-chloroacetamide (CAA) and heated at 99°C for 10 min to denature proteins. Feces were disrupted with a vortex mixer and heated again at 99°C for 5 min. The samples were centrifuged (2500g, 5min) to pellet insoluble materials. The supernatant was centrifuged again (17000g, 60−120min) to pellet bacteria. The collected supernatants were fractionated to obtain proteins using an Empore reverse-phase C-8 cartridge with 40% and 60% acetonitrile (ACN) + 0.5% acetic acid. The solutions were then evaporated using a vacuum centrifuge. The dried protein fractions were resuspended in 2M GuHCl solution, containing 10 mM TCEP and 20 mM CAA, and heated at 99°C for 5 min under agitation. The concentrations of resultant protein solutions were measured by Bradford assay. Protein solutions were then digested with 0.2 µg LysC (Promega, Sweden) for 1.5 h at 37°C under agitation and then diluted with 25 mM Tris in 10% ACN to the final concentration of 0.6 M GuHCl. Protein solutions were digested again using 0.8 µg trypsin (Promega, Sweden) overnight at 37°C under agitation. Digested solutions were acidified by 10% trifluoroacetic acid (TFA) and centrifuged to pellet any non-protein components. Peptides in the supernatant were purified and desalted using in-house made StageTips with C18 membrane (Rappsilber et al., 2007).

Samples were eluted from the StageTips using 20 μL 40% ACN in water and then 10 μL 60% ACN into a 96 well MS plate. Samples were placed in a vacuum centrifuge at 40°C until approximately 3 μL of the solution was left and then rehydrated with 10 μL of 0.1% TFA and 5% ACN solution.

Five microliters of each sample were then separated on a 15 cm column (75 μm inner diameter) which is made in-house, laser pulled, and packed with 1.9 μm C18 beads (Dr. Maisch, Germany) on an EASY-nLC 1200 (Proxeon, Odense, Denmark). The column was connected to a Q-Exactive HF-X (Thermo Scientific, Germany) on a 77 min gradient. Buffer A was milliQ water, and the peptides were separated with increasing buffer B (80% ACN and 0.1% formic acid), going from 5% to 30% in 50 min, 30% to 45% in 10 min, 45% to 80% in 2 min, held at 80% for 5 min before dropping back down to 5% in 5 min and held for 5 min. The flow rate was 250 nL/min. The column temperature was kept at 40°C. A wash-blank method using 0.1% TFA and 5% ACN solution was run in between each sample to hinder cross-contamination.

The Q-Exactive HF-X was operated in data dependent top 12 mode. Spray voltage was 2 kV, S-lens RF level was 40, and capillary was heated at 275°C. Full scan mass spectra were recorded at a resolution of 120,000 at m/z 200 over the m/z range 350–1400 with a target value of 3e6 and a maximum injection time of 25 ms. HCD-generated product ions were recorded with a maximum ion injection time set to 54 ms and a target value set to 2e5 and recorded at a resolution of 30,000. Normalized collision energy was set at 28% and the isolation window was 1.2 m/z with the dynamic exclusion set to 20 s.

**1.3 Data analysis**

RAW data files generated by LC-MS/MS were searched against a database consisting of the rhesus macaque (*M. mulatta*) proteome downloaded from Uniprot (as of 2019-06-28) and bacteria and food (i.e., rice, soybean, maize, wheat, peanuts, and sweet potato) proteomes downloaded from Swiss-Prot (as of 2019-06-20), as well as a common laboratory contaminant database provided with the MaxQuant software version 1.5.3.30 (Cox and Mann, 2008)*.* Although “fish” is also included in the ingredients of the monkey pellet, considering the supplier’s product information, its taxonomic and tissue origins are not known (i.e., industrial secret). Because the inclusion of fish proteomes originating from a number of fish species greatly enlarges the search space, fish proteins were not considered in this study. All bacterial entries in Swiss-Prot were used because no reference dataset of fecal or intestinal bacteria has been established (Lee et al., 2017). The following parameters were used for the analysis. Parent mass error and fragment mass tolerances were those pre-set for Orbitraps. Carbamidomethylation was set as a fixed modification, and oxidation of methionine, deamidation of Asparagine and Glutamine, and derivation of pyroglutamic acid were set at variable modifications. Up to a maximum of 5 modifications per peptide was allowed. All peptides were automatically filtered by a false discovery rate (FDR) of 1.0% and manually filtered by at least 2 different non-overlapping peptides. All contaminant accessions (i.e., keratins and trypsin) were excluded from further analysis using the contamination.fasta provided by MaxQuant, which includes common laboratory contaminants.

The proteome of rhesus macaque milk reported in Beck et al. (2015) was used as a comparative dataset. The RAW files reported in Beck et al. (2015) were reanalyzed using MaxQuant with the same parameters described above but searched against only the rhesus macaque proteome. Protein groups that have at least 2 unique peptides were considered as present.

Detected proteins were classified using PANTHER database version 14.1 (Mi et al., 2013). All statistical analysis was performed on R software, version 3.6.1 (R Core Team, 2019).

**2. Supplementary Results and Discussion**

**2.1 Proteins that were found from experimental blanks**

Several protein groups of macaque (e.g., keratin, neutrophil defensin 2, and protein S100) and bacteria (i.e., lysyl endopeptidase of *Pseudomonas aeruginosa* and elongation factor Tu of *Eubacterium eligens*) that are not listed as laboratory contaminants are also detected in experimental blanks (Supplementary Table 3). Although it is possible that these protein groups are contaminants, their numbers of detected peptides from experimental blanks were substantially lower than those from samples, except for lysyl endopeptidase of *P. aeruginosa*. The lysyl endopeptidase would originate from rLysC (Promega), derived from *P. aeruginosa,* that was used together with trypsin for protein digestion in this study and thus this protein group is excluded from further analysis. The other protein groups were still included in the analysis, but should be treated with caution. The protein groups that were described in the Result and Discussion sections were not detected from the experimental blanks. It is possible that the carryover of abundant peptides across the injection blank in the LC column resulted in the detection of some peptides in experimental blanks (Hendy et al., 2018).

**2.2 Potential protein marker of breast milk intake**

Protein groups that were detected in the fecal samples and those of rhesus macaque milk (Beck et al., 2015) were compared to detect potential marker protein groups for breast milk consumption (Figure 3). Among the total of 324 macaque protein groups identified from the milk (Supplementary Table 2), 88 protein groups (27.2%) were also identified in the feces. There are 17 protein groups that are shared between milk and feces and exclusively identified in breastfed individuals (i.e., MF06, MF07, MF10, and MF11), which could be potential protein markers for the detection of breast milk consumption. Nine out of the 17 protein groups were identified from at least 2 fecal samples, and all four milk-specific proteins were included here. The remaining 5 protein groups are consist of lactoperoxidase (LPO), perilipin 2 (PLIN2), fatty acid binding protein 3 (FABP3), and 2 uncharacterized proteins (Table 2; Supplementary Figure 4). LPO is contained in secretory fluids such as breast milk, saliva, and tears (Gothefors and Marklund, 1975). It catalyzes the oxidation of inorganic and organic substrates under the presence of hydrogen peroxide, and products of thiocyanate oxidized by LPO have anti-microbial activities (Gothefors and Marklund, 1975). PLIN2 is the most abundant protein on the surfaces of adipocyte lipid droplets and plays an important role in the formation and stabilization of lipid droplets (Brasaemle, 2007). It is included in the bovine milk exosome and milk fat globule membrane (Reinhardt et al., 2012). FABP3 is expressed ubiquitously but mostly in the heart and mammary glands and relates to the metabolism of long chain fatty acids (Haunerland and Spener, 2004). The other 2 uncharacterized proteins contain immunoglobulin-like domains. LPO, PLIN2, and FABP3 are all present with a relatively higher concentration in macaque breast milk than other body fluids (Beck et al., 2015) and thus could be potential protein markers of breast milk consumption in feces, although they are also expressed in other tissues.

**2.3 Details of the detected anti-microbial proteins**

Breast milk contains a wealth of anti-microbial proteins (Goldman and Goldbaum, 1995; Lönnerdal, 2003), and 12 protein groups that work as direct-acting anti-microbial agents were detected from the fecal samples analyzed in this study (Table 2; Supplementary Figure 6). Immunoglobulins, especially secretory immunoglobulin A (IgA), are the major anti-microbial proteins secreted in breast milk (Goldman and Goldbaum, 1995; Lönnerdal, 2003). Secretory IgA is a dimeric IgA complex, where the heavy chain of IgA is inter-connected by a protein called joining chain (JCHAIN), and secreted from epithelial cells of the mammary gland to breast milk through binding with polymeric immunoglobulin receptor (PIGR) (Baker et al., 2015; Kaetzel, 2005). Beta-2-microglobulin (B2M) is involved in a major histocompatibility complex and helps the immune system recognize foreign substances (Sugita and Brenner, 1994). The complement system, including C2, C3, and C9, is involved in specific and non-specific immunity and plays a major role in host defence mechanisms against infectious microbes (Korhonen et al., 2000). Finally, lactoferrin (LTF, also known as lactotransferrin: Metz-Boutigue et al., 1984), lipocalin 2 (LCN2: Golonka et al., 2019), LPO (Gothefors and Marklund, 1975), lysozyme C (LYZ: McKenzie and White, 1991), and mucin 1 (MUC1: Habte et al., 2007; Patton et al., 1994) are all involved in eliminating or killing microbes inside the digestive tract (Goldman and Goldbaum, 1995; Goldman et al., 1998; Lönnerdal, 2003).

**2.4 Confounding factors for the proteomic estimation of breastfeeding and weaning patterns**

Physiological aspects of digestion and milk components need to be considered for a valid investigation of breastfeeding and weaning patterns using fecal proteomics. If there is either a major change in the digestive ability of an individual during development, or a change in the concentration of proteins in breast milk during the entire period of breastfeeding, a change in the milk-specific proteins across different ages identified from feces does not necessarily reflect the change in breast milk consumption. Physiological and nutritional evidence suggests this is possible, but the development in digestive ability and chronological change in the concentration of proteins in breast milk seem to counteract this. An experimental study showed a comparable digestive efficiency between weaned juvenile Japanese macaques (1-year-old) and older individuals on a low-fiber diet (Sawada et al., 2012). This evidence is consistent with this study, where a similar number of food protein groups were detected from juveniles and adults (Figure 2). Unfortunately, however, there is no study that investigates a longitudinal change in the digestive ability before weaning in non-human primates, as far as we know.

Instead, experimental evidence in humans suggests that digestive ability of an individual is more than 50% that of the normal adult level at the start point of the weaning process and increases to the adult level toward the end of the weaning process. Gastric pepsin and pancreatic proteases (e.g., trypsin and chymotrypsin) are the major agents that work for the digestion of proteins (Deren, 1971; Walthall et al., 2005). Although there is no significant difference in enzymatic activity of these proteases between human infants and adults (DiPalma et al., 1991; Lindberg, 1974), the body mass-adjusted amount of secretion in these enzymes around 90−180 days after birth (i.e., the typical age at the start of weaning in humans: WHO, 2009) is at 50−90% levels compared with older children and adults (Agunod et al., 1969; Bujanover et al., 1988). The body mass-adjusted amount of secretion in digestive proteases becomes comparable to that of adults around 2 years old (i.e., the typical age at the end of weaning: WHO, 2009) in humans (Deren, 1971; Walthall et al., 2005). If a similar pattern is applicable to macaques, the digestive ability would increase to more than 50% the level of adults by 1 month old and be at a comparable level by 1 year old.

On the other hand, experimental evidence shows an increase in the proportion of proteins in macaque breast milk toward the end of the weaning period (Ôta et al., 1991; Hinde et al., 2009). In captive Japanese macaques, the proportion of protein in the total milk is approximately 1.5% around 1−2 months after birth and gradually increases to approximately 4% around 6 months (Ôta et al., 1991). A similar trend can be seen in captive rhesus macaques, and the proportion of protein in the total milk is 1.8 ± 0.03% at 1 month after birth and increases to 2.1 ± 0.04% at 3.5 months (Hinde et al., 2009).

Considering the patterns of chronological change in the digestive ability and proportion of proteins in breast milk, these physiological factors should not significantly affect an age-related pattern in the number of milk-specific proteins detected in feces. While the digestive ability experiences an approximately two-fold increase from the start to the end of weaning (Agunod et al., 1969; Bujanover et al., 1988; Deren, 1971; Walthall et al., 2005), the proportion of milk protein in breast milk also experience an approximately two-fold increase during the same period (Ôta et al., 1991; Hinde et al., 2009). These factors probably counteract each other, and a chronological pattern of the number of milk-specific proteins detected in feces would mostly reflect a degree of breast milk consumption, not systematic increases in digestive ability nor the proportion of proteins in breast milk.

**3. References**

Agunod M, Yamaguchi N, Lopez R, Luhby AL, Glass GBJ. 1969. Correlative study of hydrochloric acid, pepsin, and intrinsic factor secretion in newborns and infants. Am J Dig Dis 14:400–414.

Austin C, Smith TM, Bradman A, Hinde K, Joannes-Boyau R, Bishop D, Hare DJ, Doble P, Eskenazi B, Arora M. 2013. Barium distributions in teeth reveal early-life dietary transitions in primates. Nature 498:216–220.

Baker K, Blumberg RS, Kaetzel CS. 2015. Immunoglobulin transport and immunoglobulin receptors. In: Baker K, Blumberg RS, Kaetzel CS, editors. Mucosal Immunology: Fourth Edition. Fourth Edi. Waltham: Academic Press. p 349–407.

Beck KL, Weber D, Phinney BS, Smilowitz JT, Hinde K, Lönnerdal B, Korf I, Lemay DG. 2015. Comparative proteomics of human and macaque milk reveals species-specific nutrition during postnatal development. J Proteome Res 14:2143–2157.

Bowman JE, Lee PC. 1995. Growth and threshold weaning weights among captive rhesus macaques. Am J Phys Anthropol 96:159–175.

Brasaemle DL. 2007. The perilipin family of structural lipid droplet proteins: Stabilization of lipid droplets and control of lipolysis. J Lipid Res 48:2547–2559.

Bujanover Y, Harel A, Geter R, Blau H, Yahav J, Spirer Z. 1988. The development of the chymotryptic activity during postnatal life using the Bentiromide test. Int J Pancreatol 3:53–58.

Cox J, Mann M. 2008. MaxQuant enables high peptide identification rates, individualized p.p.b.-range mass accuracies and proteome-wide protein quantification. Nat Biotechnol 26:1367–1372.

Deren JS. 1971. Development of structure and function in the fetal and newborn stomach. Am J Clin Nutr 24:144–159.

DiPalma J, Kirk CL, Hamosh M, Colon AR, Benjamin SB, Hamosh P. 1991. Lipase and pepsin activity in the gastric mucosa of infants, children, and adults. Gastroenterology 101:116–121.

Goldman AS, Goldblum RM. 1995. Defense agents in human milk. In: Jensen R, editor. Handbook of milk composition1. San Diego: Academic Press. p 727–745.

Goldman AS, Chheda S, Garofalo R. 1998. Evolution of immunologic functions of the mammary gland and the postnatal development of immunity. Pediatr Res 43:155–162.

Golonka R, Yeoh BS, Vijay-Kumar M. 2019. The iron tug-of-war between bacterial siderophores and innate immunity. J Innate Immun 11:249–262.

Gothefors L, Marklund S. 1975. Lactoperoxidase activity in human milk and in saliva of newborn infants. Infect Immun 11:1210–1215.

Habte HH, Kotwal GJ, Lotz ZE, Tyler MG, Abrahams M, Rodriques J, Kahn D, Mall AS. 2007. Antiviral activity of purified human breast milk mucin. Neonatology 92:96–104.

Haunerland NH, Spener F. 2004. Fatty acid-binding proteins--insights from genetic manipulations. Prog Lipid Res 43:328–349.

Hendy J, Welker F, Demarchi B, Speller C, Warinner C, Collins MJ. 2018. A guide to ancient protein studies. Nat Ecol Evol 2:791–799.

Hinde K, Power ML, Oftedal OT. 2009. Rhesus macaque milk: magnitude, sources, and consequences of individual variation over lactation. Am J Phys Anthropol 138:148–157.

Hiraiwa M. 1981. Maternal and alloparental care in a troop of free-ranging Japanese monkeys. Primates 22:309–329.

Kaetzel CS. 2005. The polymeric immunoglobulin receptor: Bridging innate and adaptive immune responses at mucosal surfaces. Immunol Rev 206:83–99.

Korhonen H, Marnila P, Gill HS. 2000. Milk immunoglobulins and complement factors. Br J Nutr 84:75–80.

Lee PY, Chin S, Neoh H, Jamal R. 2017. Metaproteomic analysis of human gut microbiota: where are we heading? J Biomed Sci 24:36.

Lichtman JS, Marcobal A, Sonnenburg JL, Elias JE. 2013. Host-centric proteomics of stool: a novel strategy focused on intestinal responses to the gut microbiota. Mol Cell Proteomics 12:3310–3318.

Lindberg T. 1974. Proteolytic activity in duodenal juice in infants, children, and adults. Acta Pædiatrica 63:805–808.

Lönnerdal B. 2003. Nutritional and physiologic significance of human milk proteins. Am J Clin Nutr 77.

McKenzie HA, White FH. 1991. Lysozyme and α-lactalbumin: structure, function, and interrelationships. Adv Protein Chem 41:173–315.

Mi H, Muruganujan A, Casagrande JT, Thomas PD. 2013. Large-scale gene function analysis with the PANTHER classification system. Nat Protoc 8:1551–1566.

Ôta K, Makino Y, Kimura M, Suzuki J. 1991. Lactation in the Japanese monkey (*Macaca fuscata*): Yield and composition of milk and nipple preference of young. Primates 32:35–48.

Patton S. 1994. Detection of Large Fragments of the Human Milk Mucin MUC-1 in feces of Breast-Fed Infants. J Pediatr Gastroenterol Nutr 18:225–230.

R Core Team. 2019. R: a language and environment for statistical computing. Vienna: R Foundation for Statistical Computing.

Rappsilber J, Mann M, Ishihama Y. 2007. Protocol for micro-purification, enrichment, pre-fractionation and storage of peptides for proteomics using StageTips. Nat Protoc 2:1896–1906.

Reinhardt TA, Lippolis JD, Nonnecke BJ, Sacco RE. 2012. Bovine milk exosome proteome. J Proteomics 75:1486–1492.

Reitsema LJ, Partrick KA, Muir AB. 2015. Inter-individual variation in weaning among rhesus macaques (*Macaca mulatta*): Serum stable isotope indicators of suckling duration and lactation. Am J Primatol 78:1–22.

Sugita BM, Brenner MB. 1994. An unstable beta 2-microglobulin: major histocompatibility complex class I heavy chain intermediate dissociates from calnexin and then is stabilized by binding peptide. J Exp Med 180:2163–2171.

Tanaka I. 1992. Three phases of lactation in free-ranging Japanese macaques. Anim Behav 44:129–139.

Tsutaya T, Meaghan M, Koenig C, Sato T, Weber AW, Kato H, Olsen JV, Cappellini E. 2019. Palaeoproteomic identification of breast milk protein residues from the archaeological skeletal remains of a neonatal dog. Sci Rep 9:12841.

Walthall K, Cappon GD, Hurtt ME, Zoetis T. 2005. Postnatal development of the gastrointestinal system: A species comparison. Birth Defects Res 74:132–156.

WHO. 2009. Infant and young child feeding: Model Chapter for textbooks for medical students and allied health professionals. Geneva: World Health Organization.

**4. Supplementary Figures**

**a**


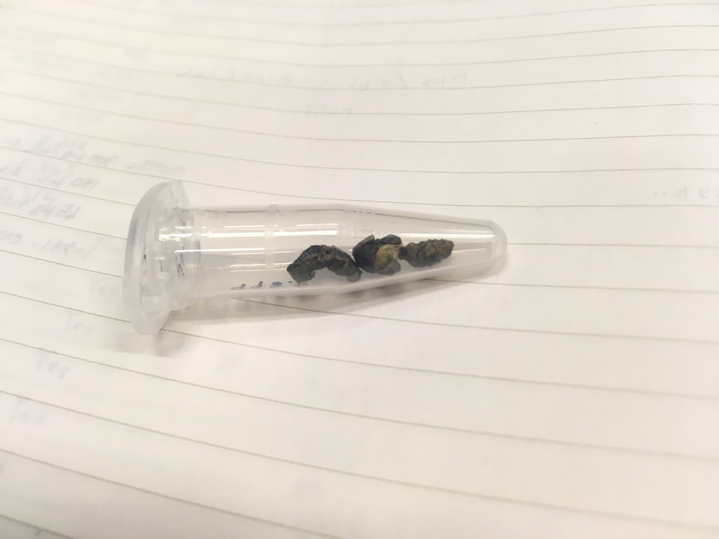


**b**


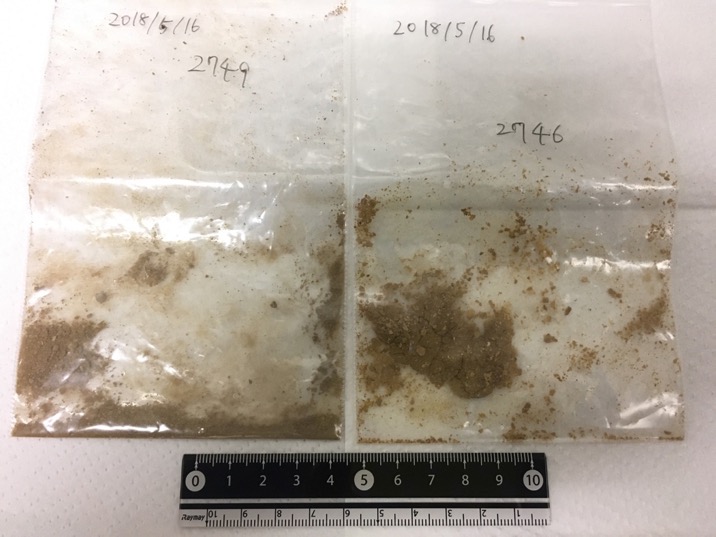


**Supplementary Figure 1.** Photo of fecal samples. **a:** meconium (MF05) and **b:** feces from non-neonate individuals. Fecal samples used in this study contained no food remain that is identifiable from visual observation.


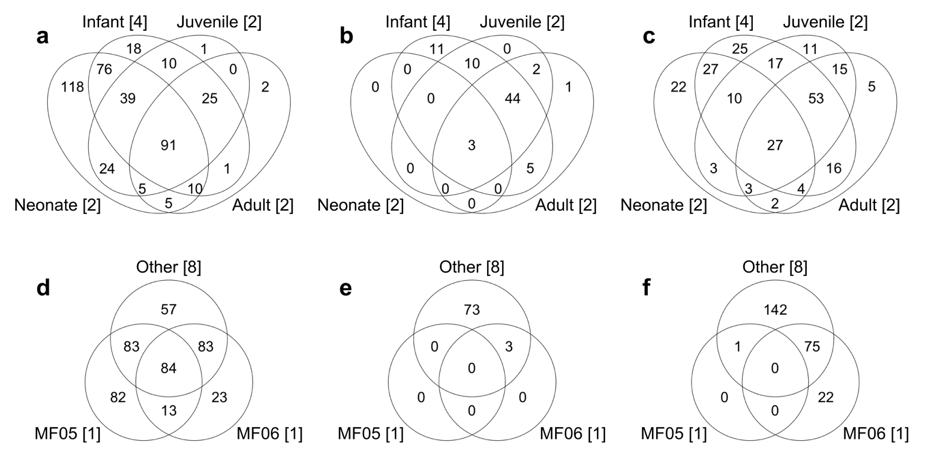


**Supplementary Figure 2.** Venn diagrams of the detected protein groups among different age categories. The number in a square bracket represents the number of samples. **a**, **b**, and **c**: comparison among neonates (MF05 and MF06), infants (MF07, MF10, MF11, and MF12), juveniles (MF03 and MF02), and adults (MF00 and MF01). **d**, **e**, and **f**: comparison among MF05 (1 day old), MF06 (36 days old), and the others. Results of macaque (**a** and **d**), food (**b** and **e**), and bacterial (**c** and **f**) proteins are shown.


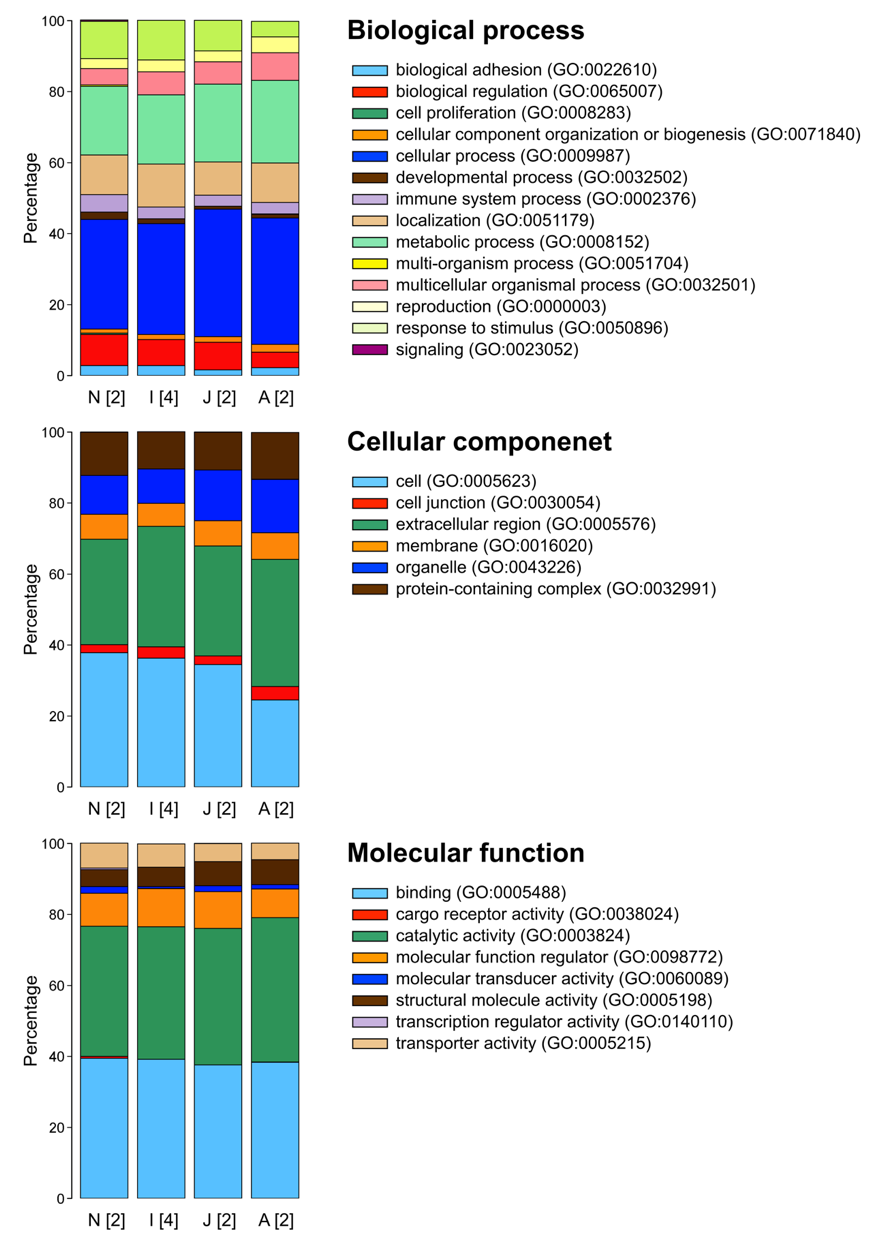


**Supplementary Figure 3.** Results of PANTHER biological process, cellular component, and molecular function analyses of protein groups that were identified in fecal samples. N, neonates (MF05 and MF06); I, infants (MF07, MF10, MF11, and MF12); J, juveniles (MF03 and MF02); and A, adults (MF00 and MF01). The number in a square bracket represents the number of samples.


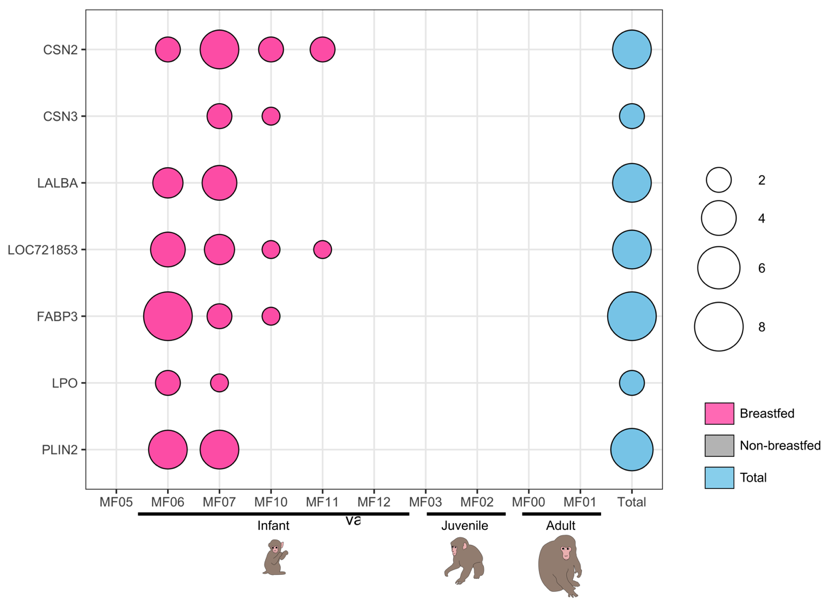


**Supplementary Figure 4.** Balloon chart showing the number of detected razor+unique peptides of milk-specific and potential breast milk marker protein groups.


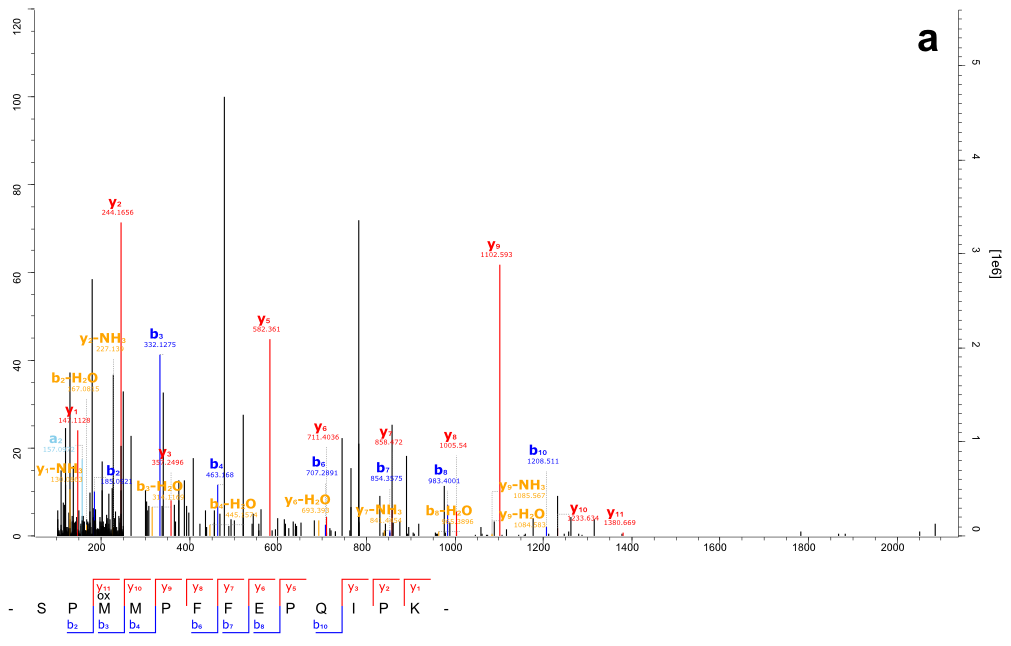

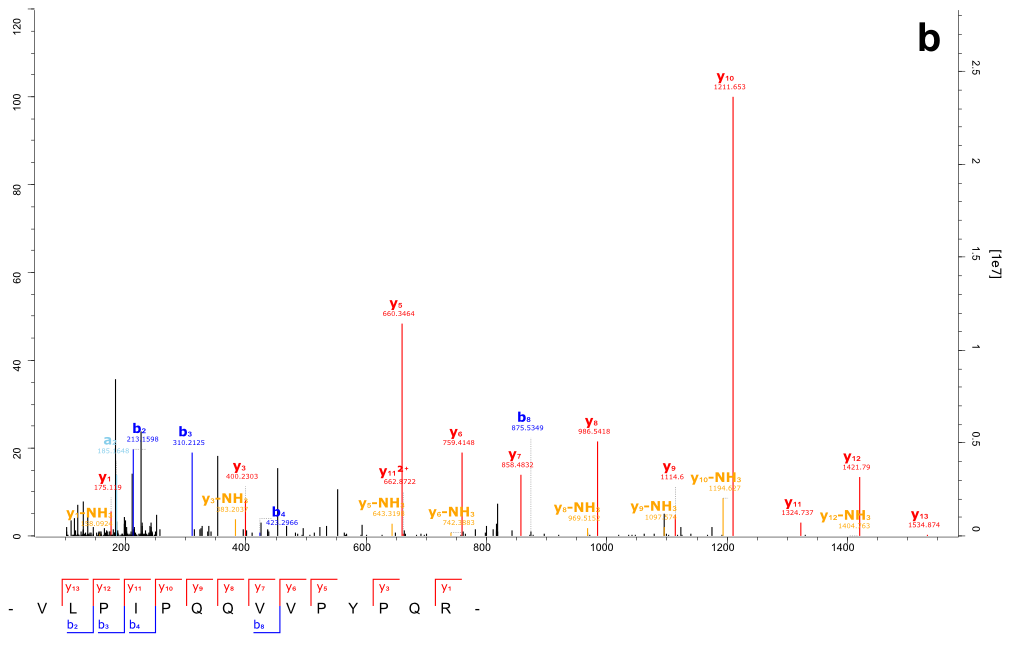

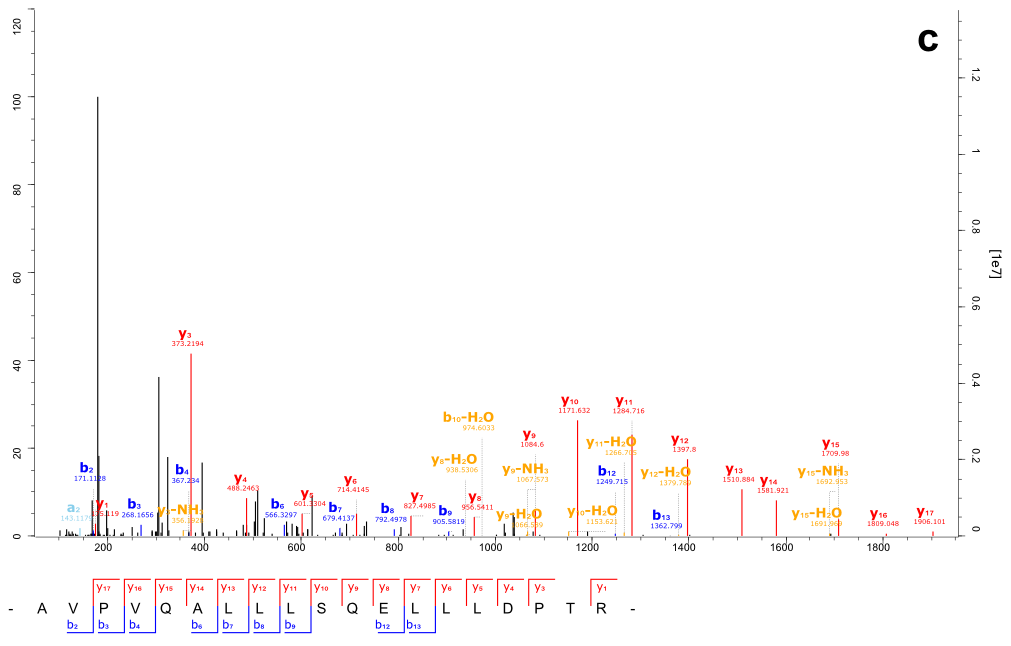

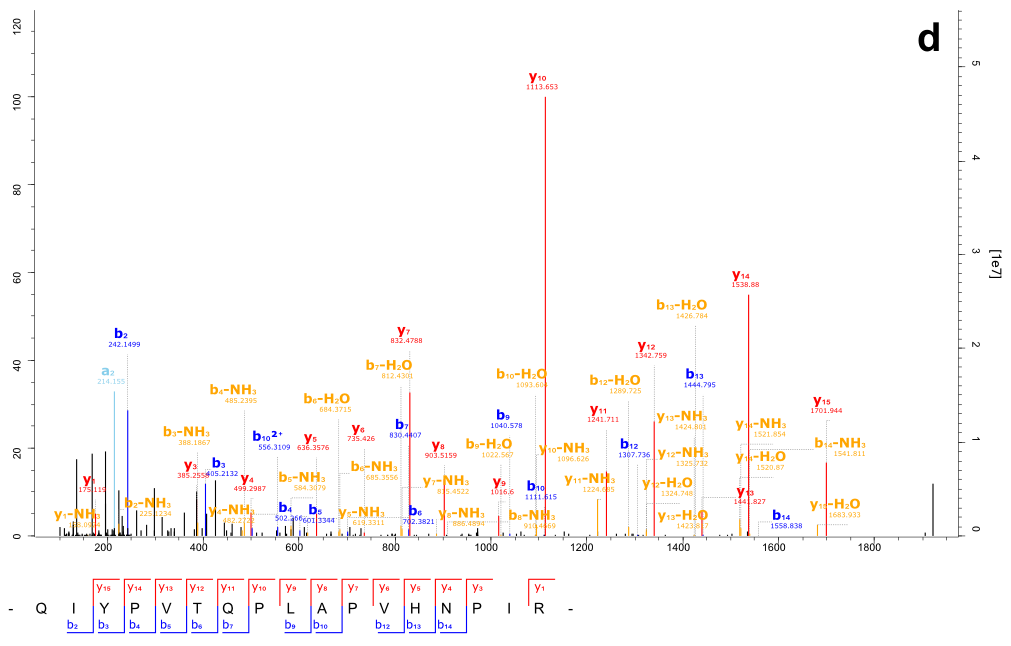

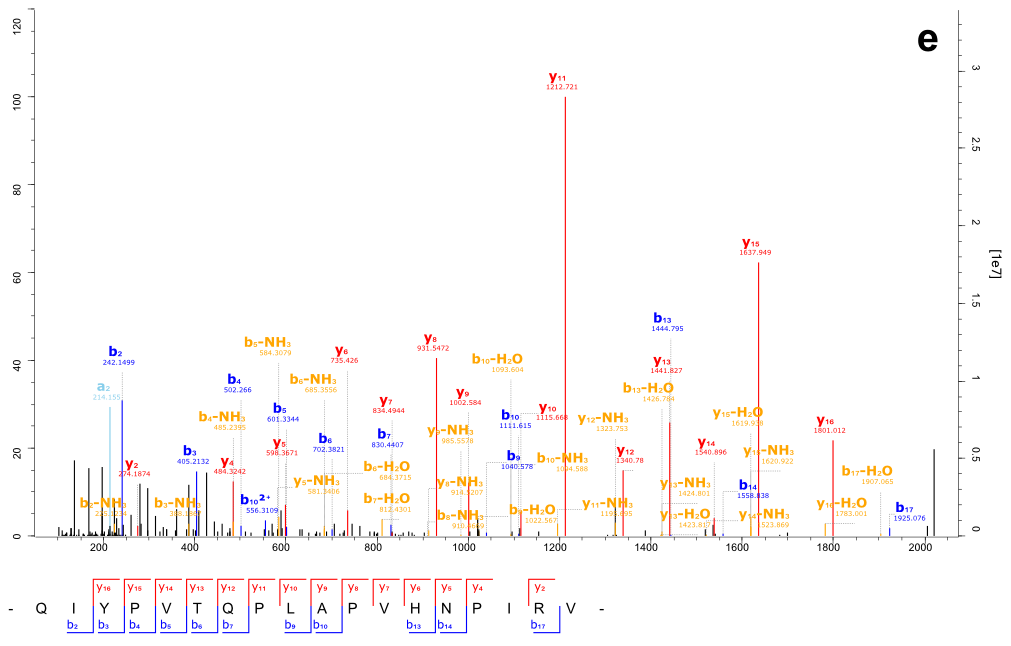

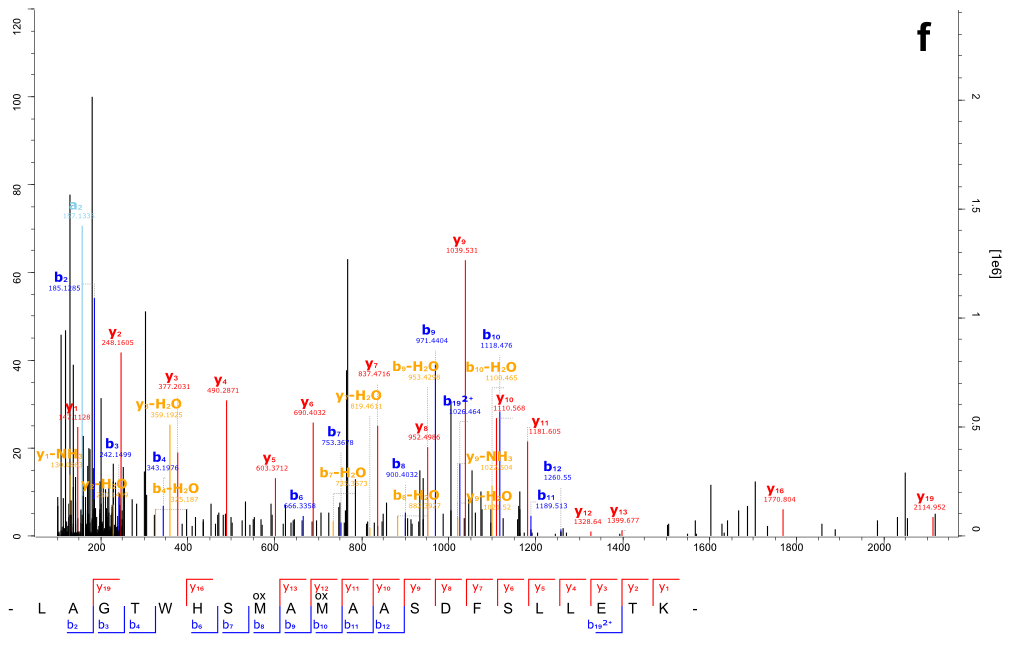

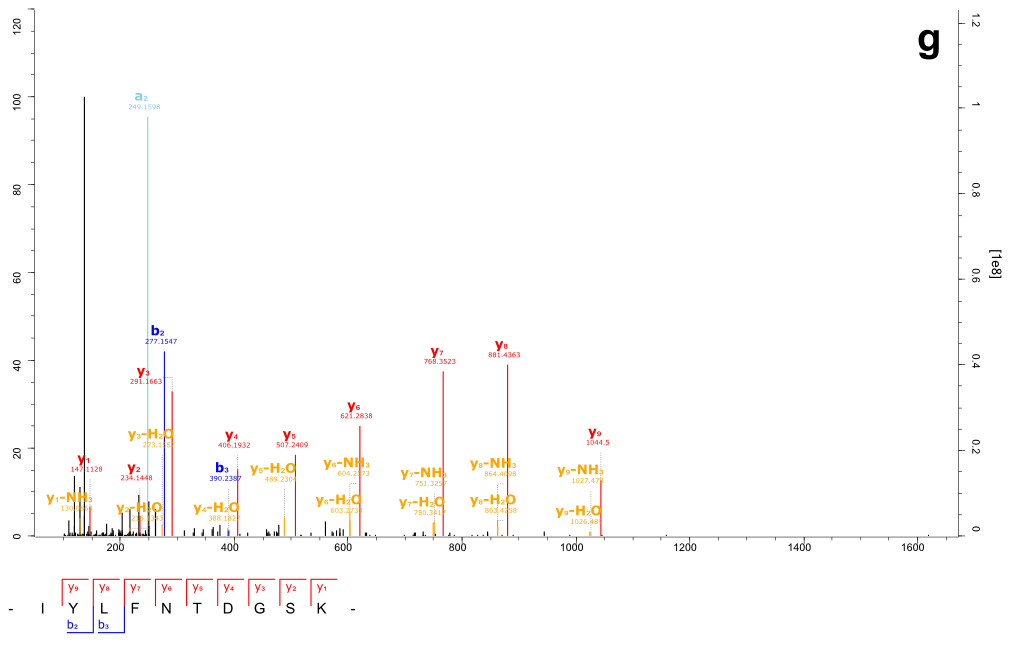

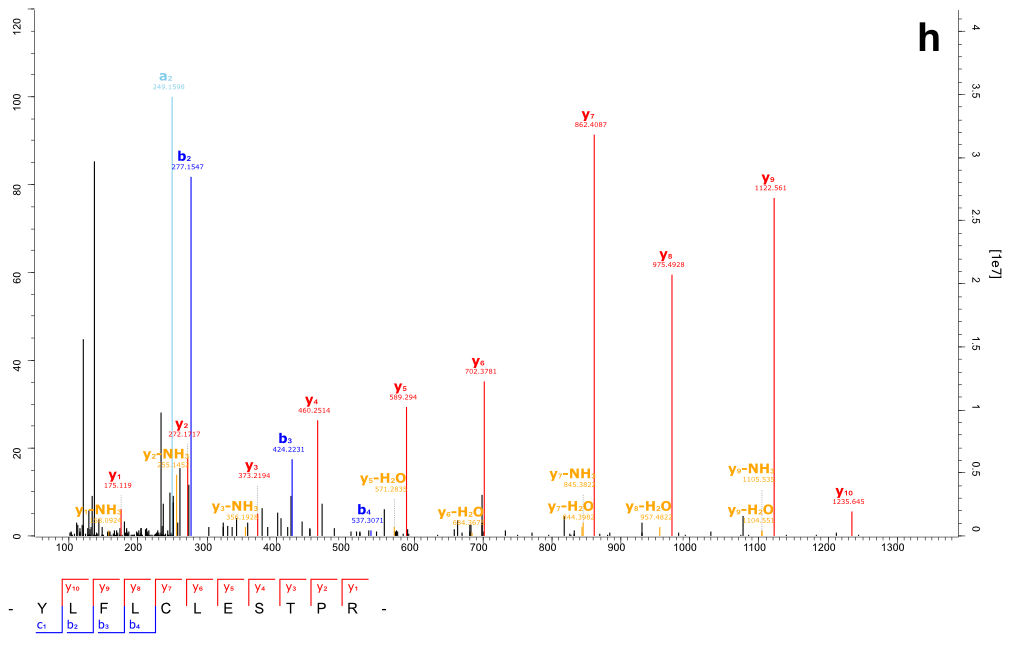

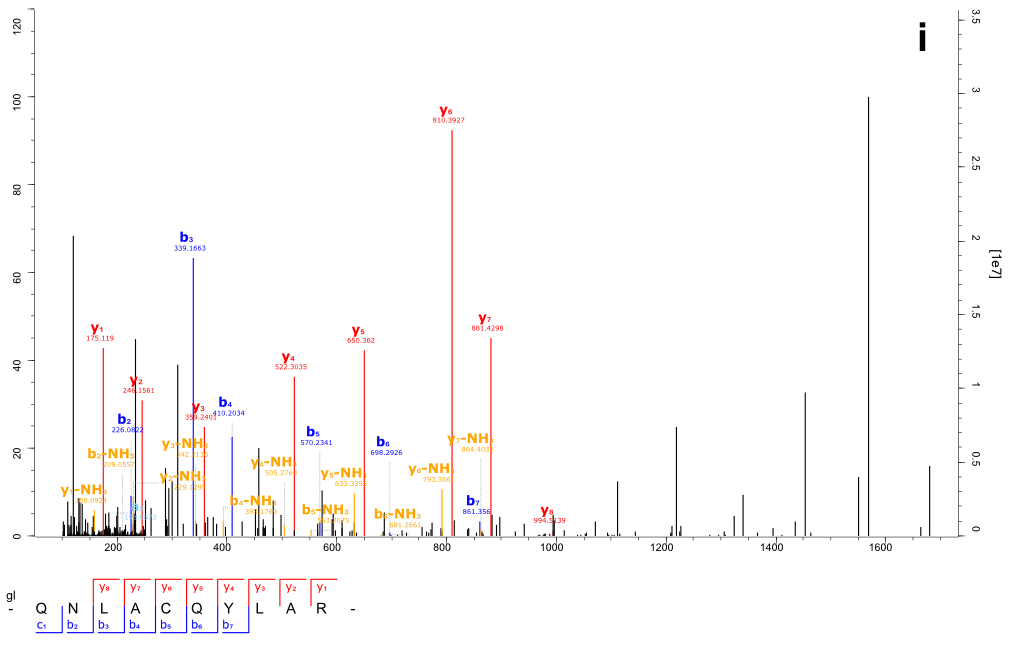

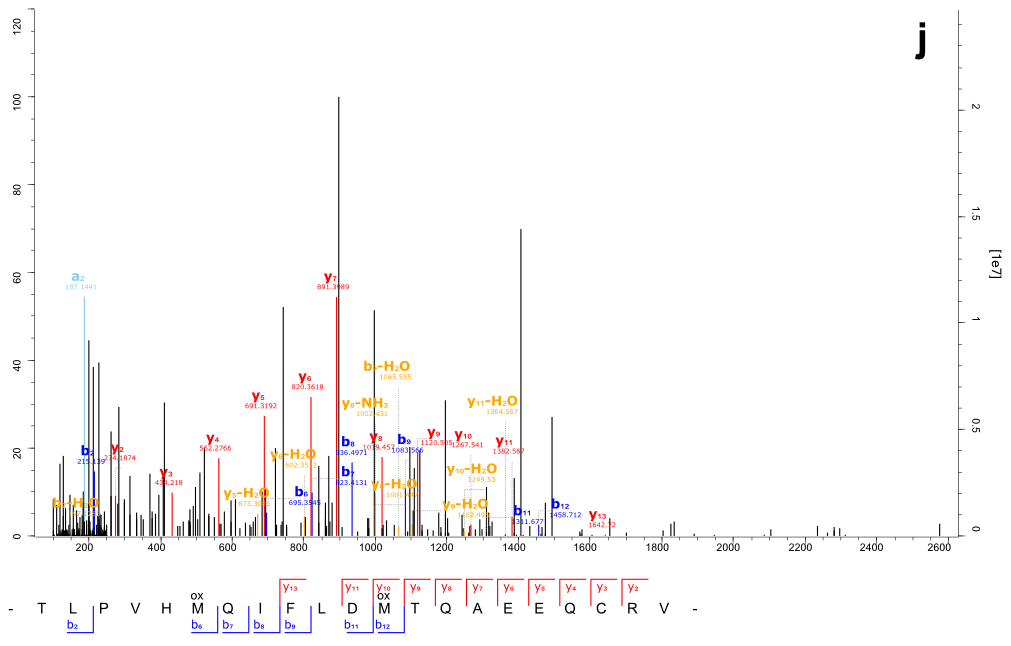

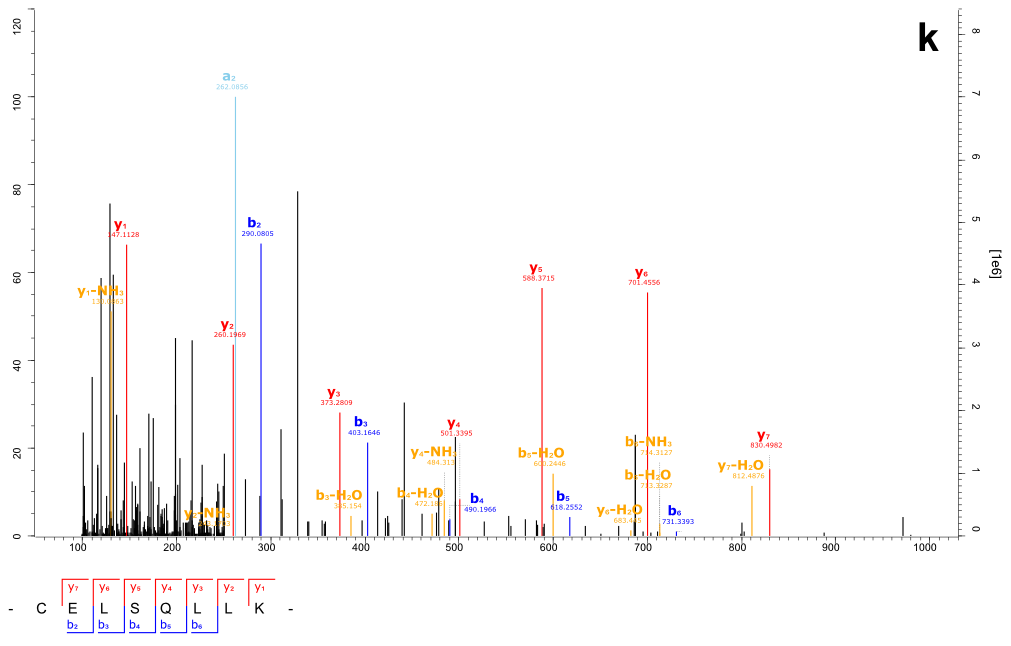

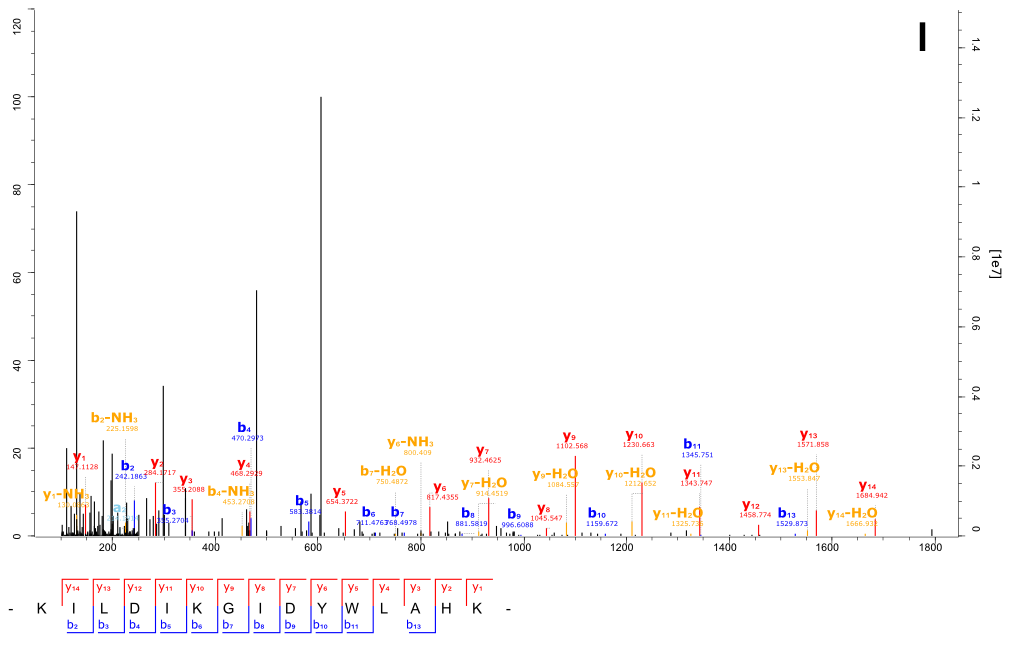

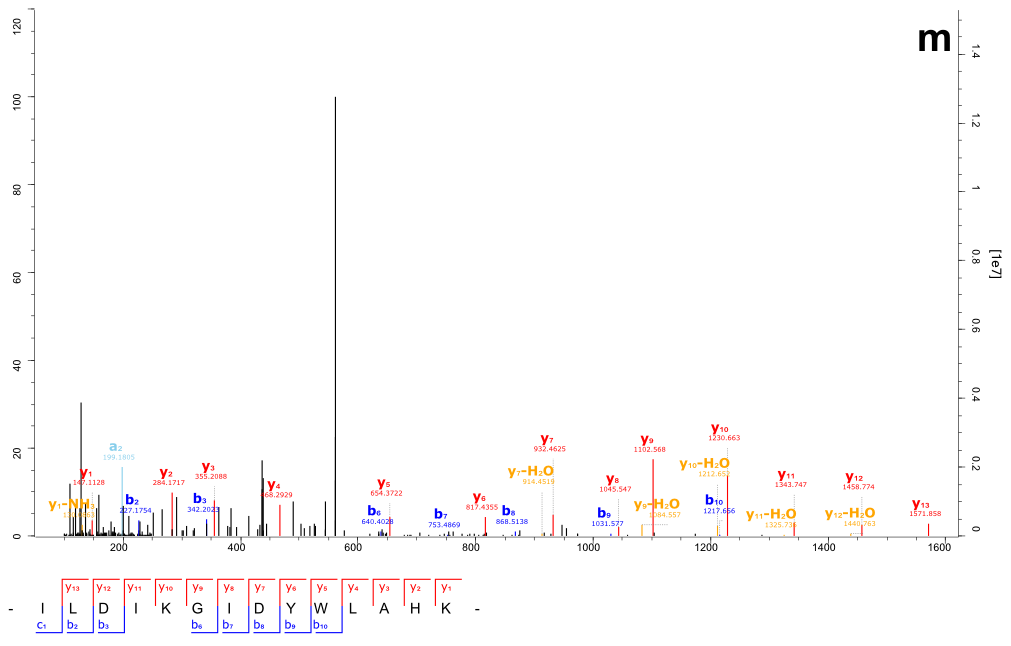

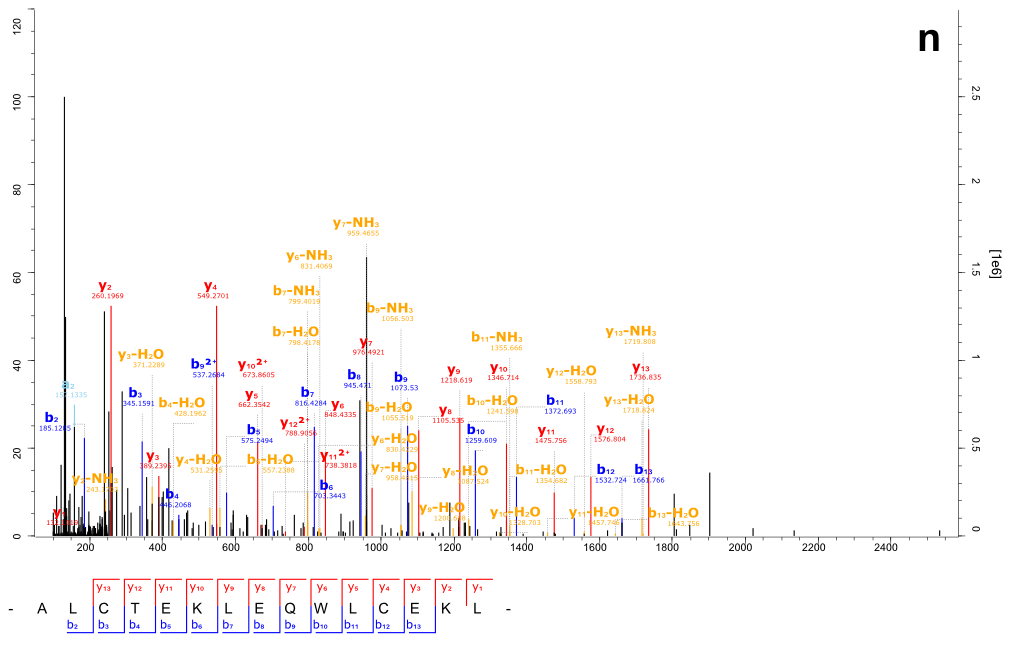

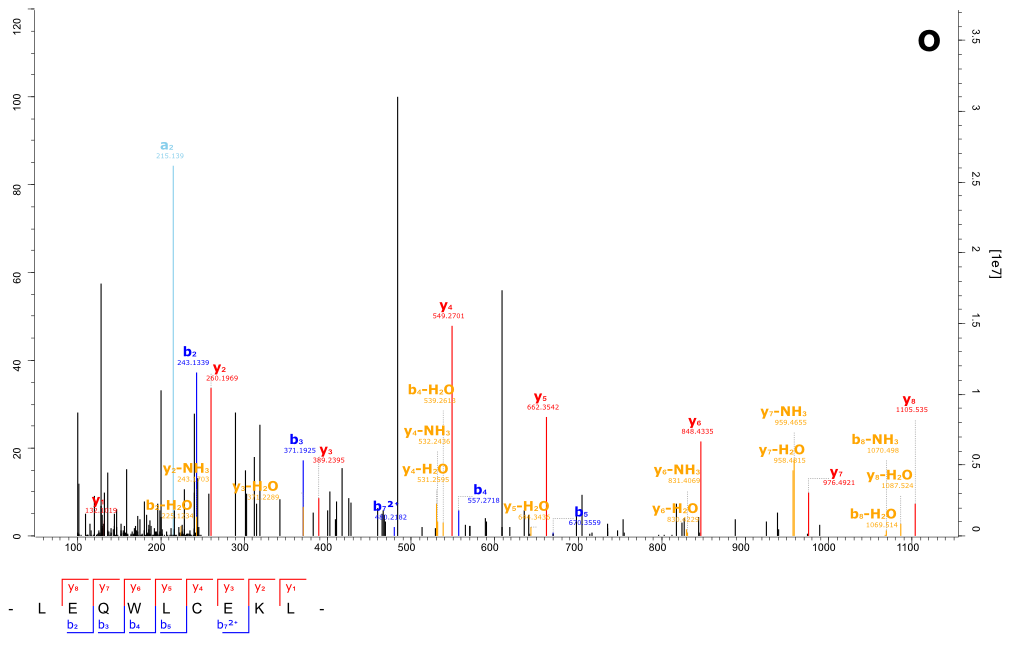

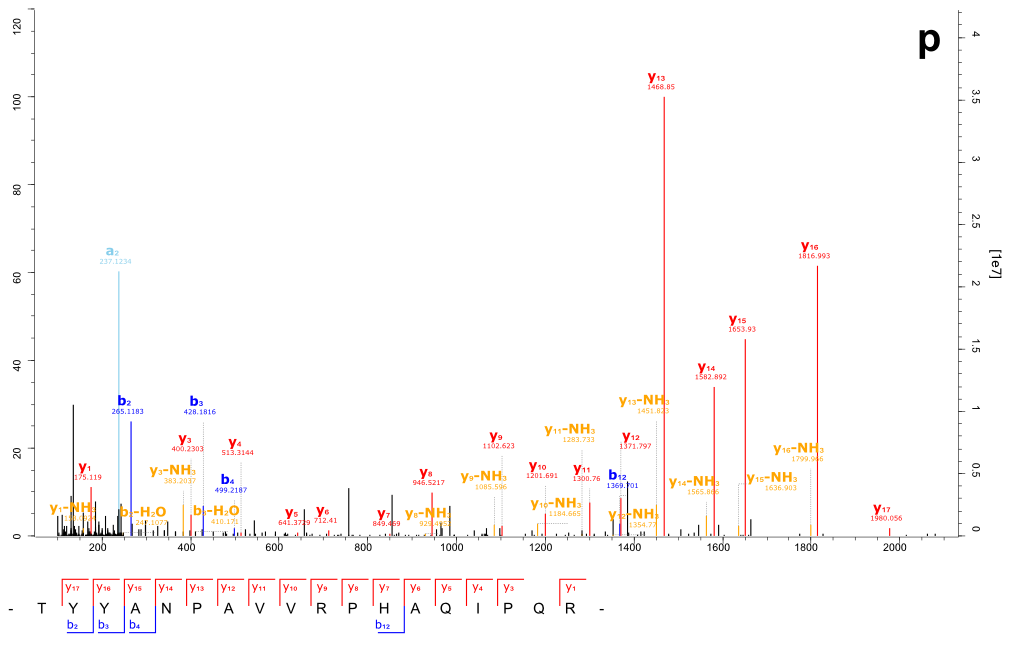

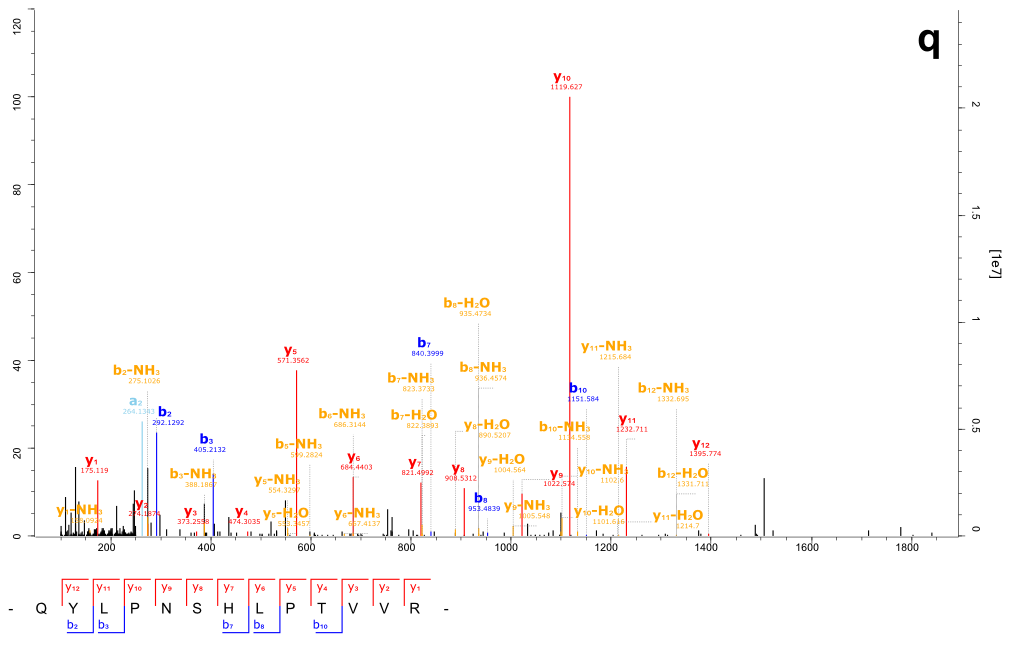


**Supplementary Figure 5.** MS2 spectra of peptides assigned to milk-specific proteins. **a-e**, CSN2; **f-j**, LOC721853; **k-o**; LALBA; and **p-q**, CSN3.


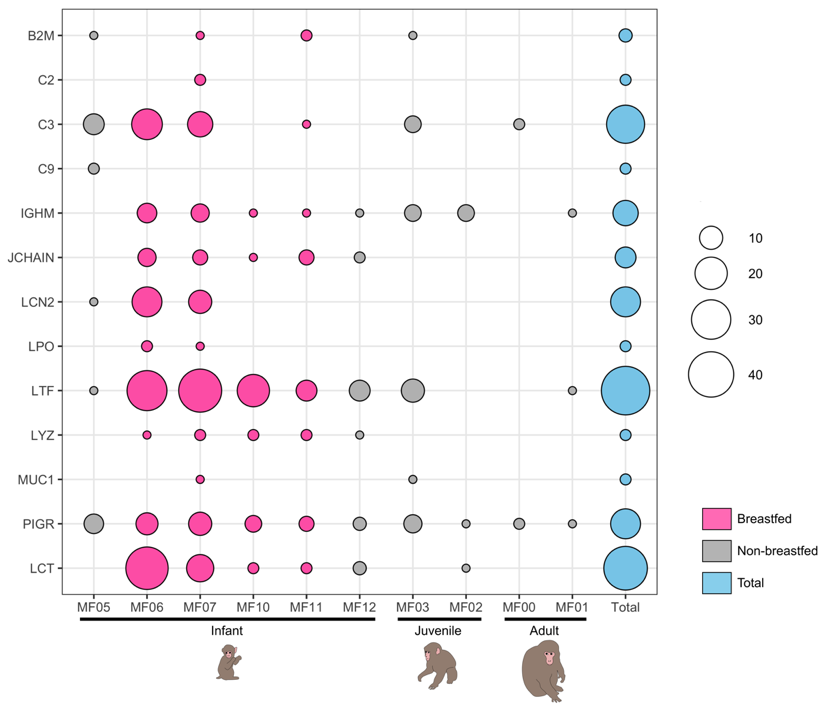


**Supplementary Figure 6.** Balloon chart showing the number of detected razor+unique peptides of anti-microbial and LCT protein groups.

**5. Supplementary Tables**

**Supplementary Table 1.** Detail of the analyzed fecal samples.

| ID | Individual | Age (Day) | Sample collection | Sample mass (mg) | Classification | Sex | Expected breastfeeding and weaning status | Milk-specific proteins |
| --- | --- | --- | --- | --- | --- | --- | --- | --- |
| MF05 | TH2729 | 1 | 2017-06-01 | 81.8 | Infant (neonate) | M | Breastfeeding is not established yet | Not detected |
| MF06 | TH2747 | 36 | 2017-09-12 | 97.8 | Infant (neonate) | F | Final period of exclusive breastfeeding | Detected |
| MF07 | TH2709 | 142 | 2017-09-12 | 61.8 | Infant | F | Intensive suckling and consumption of solid foods | Detected |
| MF10 | TH2747 | 206 | 2018-03-01 | 52.0 | Infant | F | Rapid decline in breast milk consumption | Detected |
| MF11 | TH2747 | 282 | 2018-05-16 | 65.7 | Infant | F | Breastfeeding ended or is at the final period | Detected |
| MF12 | AR2749 | 282 | 2018-05-16 | 62.3 | Infant | M | Breastfeeding ended or is at the final period | Not detected |
| MF03 | AR2622 | 756 | 2017-06-21 | 171.2 | Juvenile | M | Weaned | Not detected |
| MF02 | TH2617 | 766 | 2017-06-21 | 144.5 | Juvenile | F | Weaned | Not detected |
| MF00 | AR2239 | 3269 | 2017-06-21 | 164.0 | Adult | M | Weaned | Not detected |
| MF01 | WK2140 | 4026 | 2017-06-21 | 156.9 | Adult | F | Weaned | Not detected |

**Supplementary Table 2.** List of detected protein groups from rhesus macaque breast milk samples (Beck et al., 2015). Protein groups that are shared with Japanese macaque fecal proteome identified in this study (Supplementary Table 3) have ‘1’ in the ‘Common’ column, and otherwise ‘0’. Contaminant protein groups have a tick mark in the ‘Contamination’ column.

[supplementary_table_2.csv]

**Supplementary Table 3.** List of detected protein groups from Japanese macaque fecal samples. Protein groups that are shared with rhesus macaque milk proteome (Beck et al., 2015) have ‘1’ in the ‘Common’ column, and otherwise ‘0’. Contaminant protein groups have a tick mark in the ‘Contamination’ column. Sample names correspond with those shown in Table 1 along with the experimental blanks (MF04, MF08, and MF09).

[supplementary_table_3.csv]

**Supplementary Table 4.** Identified peptides for milk-specific proteins. Whether a peptide match (1) or not (0) to reference peptide sequences of rhesus macaque (*M. mulatta*), human (*Homo sapiens*), and cattle (*Bos taurus*) milk proteins are indicated in “Mm”, “Hs”, and “Bt” columns.

| Gene | Protein ID | Sequence | Number of missed cleavages | Start position | End position | Posterior error probability | Score | Mm | Hs | Bt |
| --- | --- | --- | --- | --- | --- | --- | --- | --- | --- | --- |
| CSN2 | F6UED1 | SPMMPFFEPQIPK | 0 | 120 | 132 | 0.0071851 | 74.133 | 1 | 0 | 0 |
|  |  | VLPIPQQVVPYPQR | 0 | 176 | 189 | 1.91E-05 | 144.1 | 1 | 1 | 0 |
|  |  | AVPVQALLLSQELLLDPTR | 0 | 190 | 208 | 4.03E-09 | 137.13 | 1 | 0 | 0 |
|  |  | QIYPVTQPLAPVHNPIR | 0 | 209 | 225 | 4.11E-202 | 270.99 | 1 | 1 | 0 |
|  |  | QIYPVTQPLAPVHNPIRV | 1 | 209 | 226 | 1.70E-125 | 241.64 | 1 | 0 | 0 |
| LOC721853 | F6YKN5 | LAGTWHSMAMAASDFSLLETK | 0 | 33 | 53 | 0.000245 | 100.53 | 1 | 0 | 0 |
|  |  | IYLFNTDGSK | 0 | 109 | 118 | 5.15E-07 | 163.64 | 1 | 0 | 0 |
|  |  | YLFLCLESTPR | 0 | 119 | 129 | 0.0041457 | 128.01 | 1 | 0 | 0 |
|  |  | QNLACQYLAR | 0 | 130 | 139 | 0.0082223 | 109.02 | 1 | 0 | 0 |
|  |  | TLPVHMQIFLDMTQAEEQCRV | 1 | 157 | 177 | 0.0083076 | 52.273 | 1 | 0 | 0 |
| LALBA | F6X0Y7 | CELSQLLK | 0 | 25 | 32 | 0.025122 | 128.12 | 1 | 1 | 0 |
|  |  | KILDIKGIDYWLAHK | 2 | 113 | 127 | 0.00062717 | 111.73 | 1 | 1 | 0 |
|  |  | ILDIKGIDYWLAHK | 1 | 114 | 127 | 0.00036706 | 112.96 | 1 | 1 | 0 |
|  |  | ALCTEKLEQWLCEKL | 2 | 128 | 142 | 1.23E-214 | 285.75 | 1 | 1 | 0 |
|  |  | LEQWLCEKL | 1 | 134 | 142 | 0.0069304 | 127.37 | 1 | 1 | 0 |
| CSN3 | F6WJ58 | TYYANPAVVRPHAQIPQR | 1 | 79 | 96 | 1.15E-16 | 154.69 | 1 | 1 | 0 |
|  |  | QYLPNSHLPTVVR | 0 | 97 | 109 | 1.61E-08 | 157.22 | 1 | 0 | 0 |

**Supplementary Table 5.** *Bifidobacterium* protein groups detected from fecal samples. Number of identified razor + unique peptides is also shown.

| Species | Gene | Protein | Protein ID | Sequence coverage | Score | N. peptide |
| --- | --- | --- | --- | --- | --- | --- |
| *Bifidobacterium adolescentis* (strain ATCC 15703 / DSM 20083 / NCTC 11814 / E194a) | rpmB | 50S ribosomal protein L28 | A0ZZV1 | 65.6 | 117.68 | 6 |
|  | rpmF | 50S ribosomal protein L32 | A0ZZX2 | 45.3 | 91.971 | 2 |
|  | rplS | 50S ribosomal protein L19 | A0ZZY0 | 27.3 | 12.741 | 2 |
|  | rplU | 50S ribosomal protein L21 | A0ZZY8 | 31.4 | 93.921 | 3 |
|  | rpmA | 50S ribosomal protein L27 | A0ZZY9 | 28.9 | 35.568 | 2 |
|  | rpsO | 30S ribosomal protein S15 | A1A007 | 51.7 | 323.31 | 4 |
|  | rplL | 50S ribosomal protein L7/L12 | A1A033 | 56.3 | 181.13 | 6 |
|  | rplM | 50S ribosomal protein L13 | A1A057 | 59.1 | 281.9 | 4 |
|  | rpsS | 30S ribosomal protein S19 | A1A073 | 57.6 | 87.857 | 6 |
|  | rplV | 50S ribosomal protein L22 | A1A074 | 33.6 | 105.72 | 4 |
|  | rpsQ | 30S ribosomal protein S17 | A1A078 | 43 | 177.14 | 3 |
|  | rplX | 50S ribosomal protein L24 | A1A080 | 32.4 | 12.71 | 2 |
|  | rplE | 50S ribosomal protein L5 | A1A081 | 44.7 | 64.599 | 5 |
|  | rplF | 50S ribosomal protein L6 | A1A084 | 15.1 | 14.425 | 2 |
|  | rpmD | 50S ribosomal protein L30 | A1A087 | 67.2 | 160.13 | 6 |
|  | rpmE | 50S ribosomal protein L31 | A1A0I8 | 71.4 | 323.31 | 4 |
|  | rpsL | 30S ribosomal protein S12 | A1A0S8 | 28.5 | 14.567 | 2 |
|  | rpsG | 30S ribosomal protein S7 | A1A0S9 | 62.8 | 158.74 | 10 |
|  | tuf | Elongation factor Tu | A1A0T1 | 28.8 | 277.12 | 7 |
|  | eno | Enolase | A1A143 | 11.1 | 39.754 | 3 |
|  | rpsB | 30S ribosomal protein S2 | A1A1H9 | 27.6 | 49.67 | 5 |
|  | pgk | Phosphoglycerate kinase | A1A1N3 | 7.5 | 11.358 | 2 |
|  | rplI | 50S ribosomal protein L9 | A1A3H8 | 27 | 47.72 | 3 |
| *Bifidobacterium animalis* subsp. lactis (strain AD011) | rplP | 50S ribosomal protein L16 | B8DW20 | 23.7 | 17.175 | 3 |
| *Bifidobacterium longum* (strain DJO10A) | rpmG | 50S ribosomal protein L33 | B3DPY6 | 50.9 | 74.98 | 2 |
| *Bifidobacterium longum* (strain NCC 2705) | ldh2 | L-lactate dehydrogenase 2 | P0CW93 | 10.9 | 12.388 | 2 |
|  | hup | DNA-binding protein HB1 | P17615 | 25.8 | 24.188 | 3 |
|  | rpmJ | 50S ribosomal protein L36 | Q8G3Z6 | 62.2 | 17.665 | 3 |
|  | infA | Translation initiation factor IF-1 | Q8G3Z7 | 50 | 21.829 | 3 |
|  | rpsH | 30S ribosomal protein S8 | Q8G404 | 25 | 26.386 | 3 |
|  | rpsJ | 30S ribosomal protein S10 | Q8G418 | 55.9 | 113.32 | 3 |
|  | rpsT | 30S ribosomal protein S20 | Q8G602 | 52.3 | 32.224 | 4 |
|  | hisE | Phosphoribosyl-ATP pyrophosphatase | Q8G694 | 29.9 | 18.713 | 3 |
|  | ilvC2 | Ketol-acid reductoisomerase (NADP(+)) 2 | Q8G6V1 | 19.7 | 56.585 | 6 |
|  | rpsR | 30S ribosomal protein S18 | Q8G758 | 50 | 236.06 | 5 |
